# Perturbations in nitric oxide homeostasis promote *Arabidopsis* disease susceptibility towards *Phytophthora parasitica*

**DOI:** 10.1111/mpp.13102

**Published:** 2021-07-09

**Authors:** Beimi Cui, Xiangren Ma, Yuan Li, Yu Zhou, Xiuyun Ju, Adil Hussain, Saima Umbreen, Bo Yuan, Anika Tabassum, Jibril Lubega, Weixing Shan, Gary J. Loake, Qiaona Pan

**Affiliations:** ^1^ The Key Laboratory of Biotechnology for Medicinal Plant of Jiangsu Province School of Life Science Jiangsu Normal University Xuzhou China; ^2^ Jiangsu Normal University–Edinburgh University, Centre for Transformative Biotechnology of Medicinal and Food Plants Jiangsu Normal University Xuzhou China; ^3^ Institute of Molecular Plant Sciences School of Biological Sciences University of Edinburgh Edinburgh UK; ^4^ Department of Agriculture Abdul Wali Khan University Mardan Pakistan; ^5^ State Key Laboratory of Crop Stress Biology for Arid Areas and College of Agronomy Northwest A&F University Yangling China

**Keywords:** nitric oxide, *Phytophthora parasitica*, reactive oxygen species, salicylic acid, *S*‐nitrosylation

## Abstract

*Phytophthora* species can infect hundreds of different plants, including many important crops, causing a number of agriculturally relevant diseases. A key feature of attempted pathogen infection is the rapid production of the redox active molecule nitric oxide (NO). However, the potential role(s) of NO in plant resistance against *Phytophthora* is relatively unexplored. Here we show that the level of NO accumulation is crucial for basal resistance in *Arabidopsis* against *Phytophthora parasitica*. Counterintuitively, both relatively low or relatively high NO accumulation leads to reduced resistance against *P. parasitica*. *S*‐nitrosylation, the addition of a NO group to a protein cysteine thiol to form an *S*‐nitrosothiol, is an important route for NO bioactivity and this process is regulated predominantly by *S*‐nitrosoglutathione reductase 1 (GSNOR1). Loss‐of‐function mutations in *GSNOR1* disable both salicylic acid accumulation and associated signalling, and also the production of reactive oxygen species, leading to susceptibility towards *P. parasitica*. Significantly, we also demonstrate that secreted proteins from *P. parasitica* can inhibit *Arabidopsis* GSNOR1 activity.

## Graphical abstract

Perturbations in *Arabidopsis* nitric oxide homeostasis promote disease susceptibility towards the oomycete pathogen *Phytophthora parasitica*.

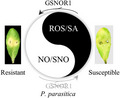

## 1 INTRODUCTION

The genus *Phytophthora* contains some of the most destructive species of plant oomycetes that infect hundreds of different plant species, including many trees and crops, causing a number of serious and agriculturally significant diseases (Grünwald et al., 2012; Jung et al., 2016; Nowicki et al., 2012). For example, the late blight disease caused by *Phytophthora infestans* triggered the great Irish famine from 1845 to 1849 (Fisher et al., 2012). Because oomycetes exhibit a fungus‐like morphology, they were originally classified as fungi; however, recent evolutionary analysis has placed them into a separate kingdom termed Stramenopila (Van de Peer & De Wachter, 1997). Oomycete pathogens have evolved a sophisticated system to avoid the host immune response (Kamoun et al., 2015; Latijnhouwers et al., 2003). Thus, it is difficult to control *Phytophthora* diseases. Uncovering the mechanisms by which *Phytophthora* species infect their target plants and the associated immune responses deployed by their hosts may provide new strategies for disease control against these economically significant pathogens.

It has been reported that *rph1* (resistance to *Phytophthora* 1), which encodes a chloroplast protein, showed increased susceptibility to *P*. *brassicae* infection by affecting the reactive oxygen species (ROS) burst in response to *Phytophthora* infection. Furthermore, the function of RPH1 in resistance to *P*. *brassicae* is conserved in both *Arabidopsis* and potato (Belhaj et al., 2009). *Arabidopsis* plants missing the L‐type lectin receptor kinase (LecRK) showed increased susceptibility to both *Phytophthora* *brassicae* and *Phytophthora capsici* (Wang et al., 2014). In addition, *RTP1* (*Arabidopsis thaliana* Resistant to *Phytophthora* 1), encoding a novel endoplasmic reticulum‐localized protein, and *AtRTP5* (*Arabidopsis thaliana* Resistant to *Phytophthora* 5), which encodes a WD40 repeat domain‐containing protein, negatively regulate plant resistance to *Phytophthora* (Li, Zhao, et al., 2020; Pan et al., 2016), highlighting that the interactions between *Phytophthora* and their host plants are highly complex.

A major strategy for microbial pathogens to overcome plant immune systems is by the secretion of effector proteins, which interfere with defence responses, thereby enhancing infection (Dodds & Rathjen, 2010; Jones & Dangl, 2006). *Phytophthora* species carry hundreds of such effector proteins (Wang et al., 2019). For example, an RXLR effector, *SFI5*, from *P. infestans* interferes with early immune responses including the ROS burst and the expression of key defence genes (Zheng et al., 2014). Furthermore, eight of 34 examined effectors from *P. infestans* suppress immune responses in tobacco, indicating that *Phytophthora* effectors target key elements of the defence system during infection to aid colonization (Zheng et al., 2014). In this context, PcAvh103 and RxLR48 from *P. capsici* interacted with enhanced disease susceptibility 1 (EDS1) and nonexpressor of PR1 (NPR1), respectively, both key regulatory nodes in the plant defence signalling network, promoting pathogen virulence by disrupting signalling via the immune activator, salicylic acid (SA) (Li, Chen, et al., 2019; Li, Wang et al., 2020).

A key feature of attempted pathogen infection is the rapid production of small, redox active molecules, including nitric oxide (NO) and ROS. These redox molecules orchestrate a plethora of immune responses in plants, including the accumulation of SA (Feechan et al., 2005; Grant & Loake, 2000; Lindermayr et al., 2010; Tada et al., 2008). A major route for the transfer of NO bioactivity is *S*‐nitrosylation, the covalent attachment of NO to a cysteine thiol (SH) to form an *S*‐nitrosothiol (SNO) (Jahnová et al., 2019; Yu et al., 2014). This redox‐based posttranslational modification (PTM) is established with exquisite specificity (Astier et al., 2011; Umbreen et al., 2018), largely due to the unique properties of the sulphur atom component of the cysteine thiol (Umbreen et al., 2018). The enzyme *S*‐nitrosoglutathione reductase 1 (GSNOR1) is a key determinant in indirectly controlling the total levels of cellular *S*‐nitrosylation by depleting *S*‐nitrosoglutathione (GSNO), the major cellular NO donor (Chen et al., 2009; Feechan et al., 2005; Lee et al., 2008). *Arabidopsis* GSNOR1 is required for multiple modes of plant disease resistance (Feechan et al., 2005) and also some aspects of plant development (Kwon et al., 2012; Lee et al., 2008) and this function is conserved in tomato (Gong et al., 2019; Hussain et al., 2019; Matamoros et al., 2020). However, a potential role for GSNOR1 in resistance to *Phytophthora* infection has not been uncovered.

It has recently been demonstrated, however, that exogenous application of the NO donor sodium nitroprusside (SNP) reduced resistance against *Phytophthora* (El‐Beltagi et al., 2017). Moreover, ROS and SA accumulation are also thought to be required for resistance against this pathogen in both tobacco and *Arabidopsis* (Li, Wang, et al., 2020; Pan et al., 2016; Wi et al., 2012). In this context, catalase2 (CAT2) is directly targeted by PsCRN63 from *P. sojae*, leading to increased H_2_O_2_ levels, triggering plant cell death (Zhang et al., 2015). SA signalling is also thought to be targeted by the *Phytophthora* *sojae* effector PsICS1 and the *P. capsici* effector RxLR48 (Li, Chen, et al., 2019; Liu et al., 2014). However, evidence that effectors might manipulate immune‐related redox signalling remains to be established.

In this study, we employed the *Arabidopsis–Phytophthora* pathosystem to explore the role of redox signalling in this interaction. Our findings suggest that NO accumulation within a given concentration range supports *Arabidopsis* basal resistance against *P. parasitica*. Furthermore, GSNOR1 is required for full basal resistance against this pathogen. Transcriptomic and associated genetic analysis suggested that *gsnor1* plants have impaired SA signalling and ROS production, leading to enhanced susceptibility to *P. parasitica*. Significantly, we have also demonstrated that secreted proteins from *P. parasitica* may inhibit GSNOR1 activity.

## 2 RESULTS

### 2.1 NO is required for basal disease resistance of *Arabidopsis* against *P. parasitica*

**FIGURE 1:**
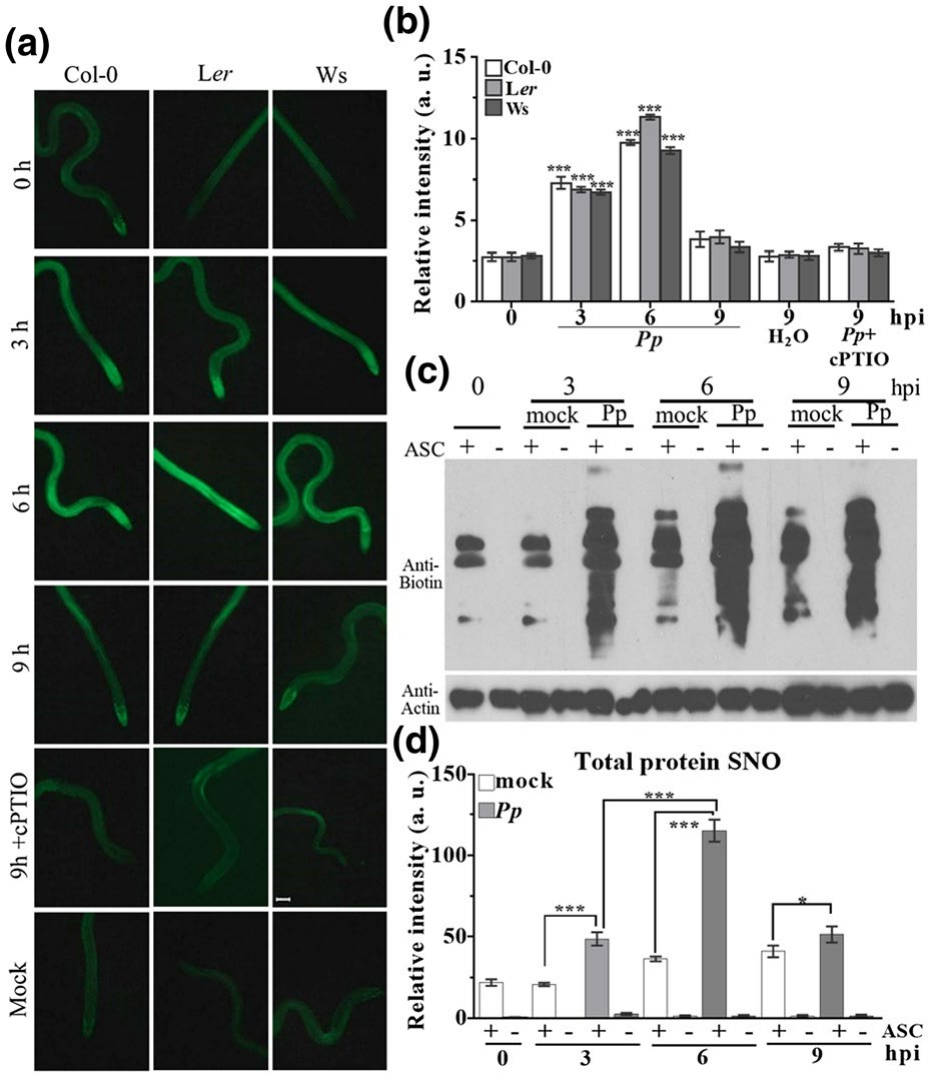
*Phytophthoraparasitica* infection elevates (S)NO levels in *Arabidopsis*. (a, b) NO levels determined in given genotypes by DAF‐FM staining. Roots of living plants after *P. parasitica* infection or mock treatment were stained with DAF‐FM DA and photographed with a Leica SP5 fluorescence microscope (a) and the relative intensity of DAF‐FM stain was determined by ImageJ software (b) at indicated time points. Water was used as control. Eight seedlings of each line were used. Error bars represent the *SD* of eight replicates from each line. Asterisks indicate statistically significant differences compared to 0 hr postinoculation (hpi) (one‐way analysis of variance [ANOVA], ***p < .001). (c, d) Total SNO detection by biotin switch assay followed by quantification. An equal amount of total protein extracts was subjected to the biotin‐switch assay after *P. parasitica* infection or mock treatment. Ascorbate (ASC) was employed as negative control for SNO formation. The signal was quantified by ImageJ software. Experiments were repeated three times with similar results. *p < .05, ***p < .001, one‐way ANOVA

A key feature following pathogen recognition in eukaryotes is the engagement of a nitrosative burst, which leads to the accumulation of the gaseous signalling molecule NO and activation of cognate defence systems (Delledonne et al., 1998; Yu et al., 2014). However, the potential role of NO in basal disease resistance is not well documented. Thus, we explored if the NO burst was engaged during basal disease resistance against *P. parasitica*. *Arabidopsis* seedlings were challenged by *P. parasitica* and endogenous changes in NO levels were analysed by real‐time imaging of NO accumulation using 4‐amino‐5‐methylamino‐2',7'‐difluorofluorescein diacetate (DAF‐FM DA). The endogenous NO level in wildtype Col‐0 plants displayed a dynamic change postinoculation with a small increase at 3 hr postinoculation (hpi), together with a more significant increase at 6 hpi, followed by a decrease to unchallenged levels at 9 hpi (Figure 1a,b). Thus, NO accumulates during the early stages of basal disease resistance against *P. parasitica*. Subsequently, we checked additional *Arabidopsis* genotypes, Ler and Ws, which are also susceptible to *P*. *parasitica*. These genotypes showed a similar NO accumulation pattern in response to *P. parasitica* (Figure 1a,b). Collectively, these results suggest that *P. parasitica* can induce an NO burst in *Arabidopsis*.

A major route for the transfer of NO bioactivity is *S*‐nitrosylation, the addition of an NO moiety to a protein cysteine thiol, forming an SNO (Yu et al., 2014). Therefore, we examined total SNO levels in *Arabidopsis* after *P. parasitica* inoculation employing the biotin‐switch assay (Jaffrey & Snyder, 2001). The total SNO level was significantly increased in Col‐0 plants following *P. parasitica* inoculation compared to mock‐treated plants (Figure 1c,d). These results suggest that *P. parasitica* triggered NO accumulation and subsequent SNO generation at early stages during the deployment of basal disease resistance.

**FIGURE 2:**
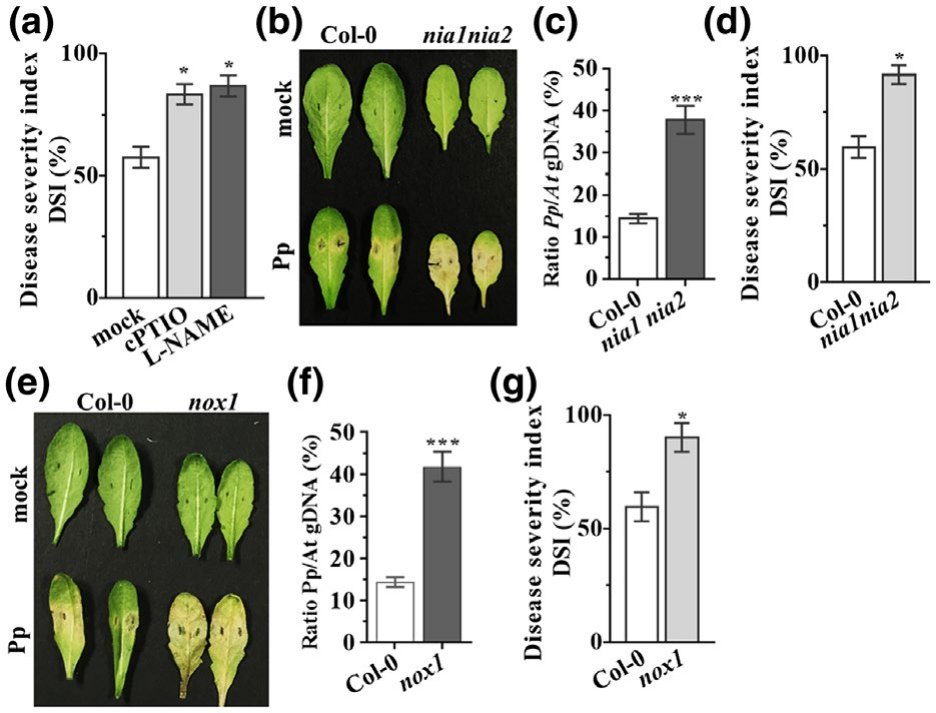
The role of nitric oxide in *Phytophthora* *parasitica–Arabidopsis* interactions. (a) Disease severity index (DSI) of Col‐0 seedlings in response to *P. parasitica* in the presence of 150 μM L‐NAME (L‐NG‐nitroarginine methyl ester) or 200 μM cPTIO (2‐4‐carboxyphenyl‐4,4,5,5‐tetramethylimidazoline‐1‐oxyl‐3‐oxide) for 4 days. (b) Phenotype of detached leaves inoculated with Pp016 for 3 days. Pp, infected by *P. parasitica*; Mock, treated by water. (c) Pathogen biomass analysis by quantitative PCR (qPCR). The DNA ratio of *P. parasitica* compared to *Arabidopsis* *thaliana* (*PpUBC*/*AtUBC9*) was determined by qPCR, total DNA extracted from inoculated leaves was used as a template. Error bars represent *SD* of six biological replicates. (d) The means of disease severity index of Col‐0 and *nia1 nia2* seedlings inoculated with Pp016. (e) Phenotype of indicated genotypes of detached leaves inoculated with Pp016. (h) Pathogen biomass analysis determined by qPCR. (f) Incidence of *P. parasitica* on wildtype Col‐0 and *nox1* seedlings 4 days postinoculation (dpi). For the mean of disease severity index assay, 16 seedlings were used for each experiment, error bars represent *SD* from three replicates. Asterisks indicate statistically significant differences compared to wildtype Col‐0. One‐way analysis of variance, ***p < .001, **p < .005, *p < .05

To further explore a possible role for the nitrosative burst in *Arabidopsis* basal disease resistance against *P. parasitica*, we employed the NO scavenger 2‐4‐carboxyphenyl‐4,4,5,5‐tetra‐methylimidazoline‐1‐oxyl‐3‐oxide (cPTIO) and the mammalian NOS inhibitor L‐NG‐nitro‐arginine methyl ester (L‐NAME), which has been shown to inhibit a NOS‐like activity in plants (Delledonne et al., 1998). The application of cPTIO and L‐NAME both enhanced the susceptibility of Col‐0 plants to *P. parasitica* (Figures 2a and S1a). We next checked the nitrate reductase (NR)‐deficient double mutant *nia1 nia2*, which has been reported to exhibit reduced NO accumulation (Modolo et al., 2006) (Figure S1b,c) to *P. parasitica* inoculation. The *nia1 nia2* line exhibited enhanced disease susceptibility to *P. parasitica* compared with Col‐0 plants (Figure 2b,d). This enhanced susceptibility phenotype was confirmed by determining the biomass of *P. parasitica* (Figure 2c). Collectively, our data suggest that NO accumulation contributes to *Arabidopsis* basal resistance against *P. parasitica*.

To further explore the contribution of NO to *Arabidopsis* basal disease resistance against *P. parasitica*, we employed the NO hyperaccumulating line, *no overexpression 1* (*nox1*). Unexpectedly, the leaves of *nox1* plants also exhibited enhanced disease susceptibility to *P. parasitica* relative to Col‐0 (Figure 2e). A biomass assay also confirmed significantly higher growth and proliferation of *P. parasitica* in *nox1* plants as compared to Col‐0 plants (Figure 2f). Counterintuitively, *nox1* seedlings exhibited an increased disease severity index relative to wildtype plants (Figure 2g), whereas application of cPTIO partially rescued this phenotype (Figure S1d). In aggregate, our data suggest that either reduced or enhanced NO levels negatively impact basal resistance against *P. parasitica*.

### 2.2 *GSNOR1* is required for basal resistance against *P. parasitica* in *Arabidopsis*

**FIGURE 3:**
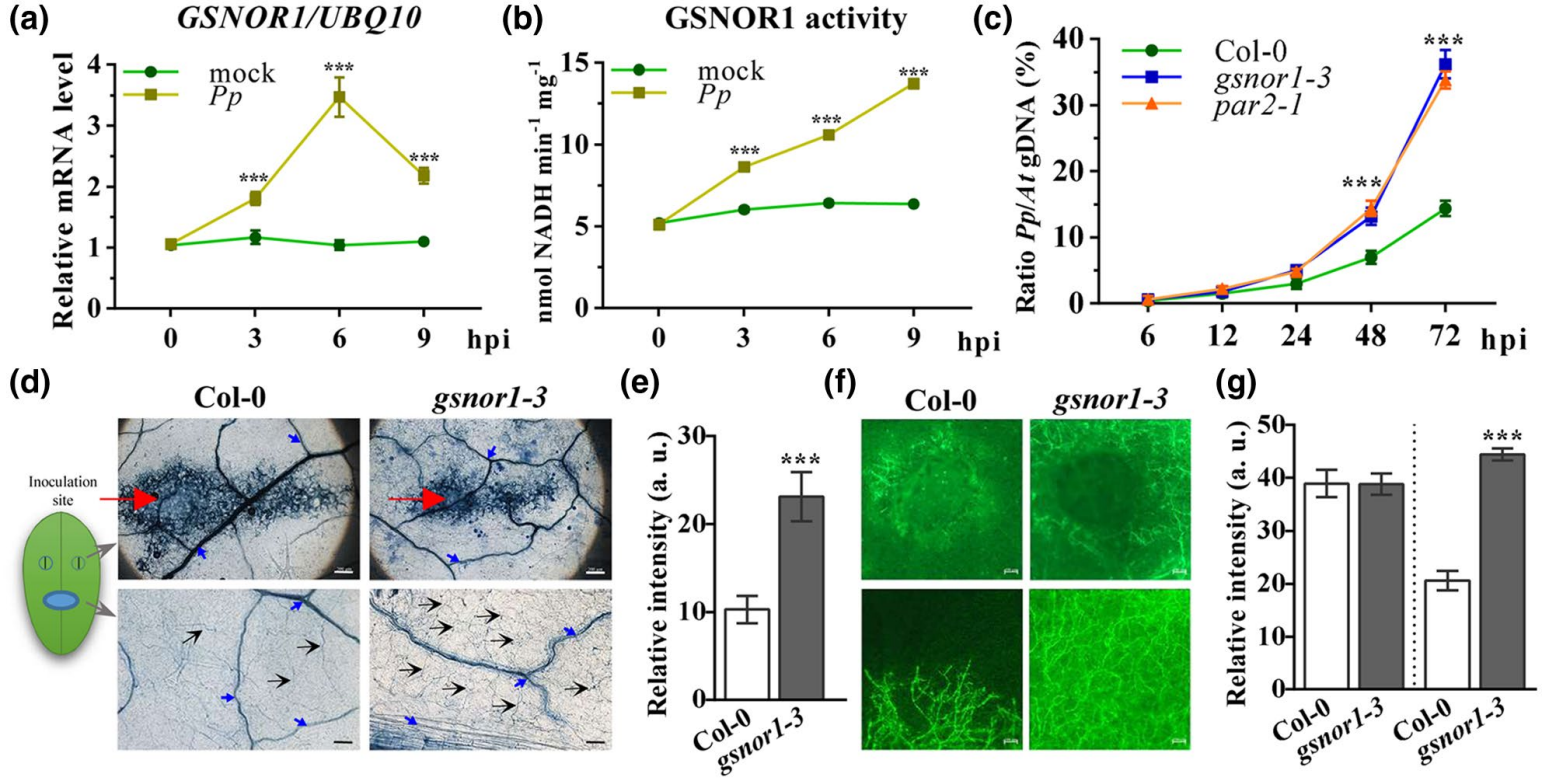
GSNOR1 is required for *Arabidopsis* resistance to *Phytophthora* *parasitica* infection. (a) *GSNOR1* transcript accumulation on infection was assayed by quantitative reverse transcription PCR. (b) GSNOR activity was measured at the indicated time points after inoculation with *P. parasitica*. (c) Pathogen biomass analysis by quantitative PCR for indicated genotypes after *P. parasitica* inoculation. Error bars in a–c indicate *SD* of three biological replicates. (d) Representative image of pathogen colonization in infected leaves by trypan blue staining. Schematic showing the corresponding observation site on the leaf: upper row (inoculation site) and lower row. Scale bars: upper row, 200 μm; lower row, 100 μm. Red arrow: wound site to enable colonization. Black arrow: hypha. Blue arrow: plant vein. (e) Relative intensity quantification of trypan blue staining in lower row of (d). Error bars represent *SD* of eight replicates. (f) Representative images of green fluorescent protein (GFP) expression *P. parasitica* H1121 hyphae colonization in detached leaves 3 days postinoculation. Upper row shows inoculation site, while lower row represents the middle of infected leaves. Scale bars: 100 μm. (g) Relative intensity of GFP signal of (e) was determined by ImageJ software. Error bars represent *SD* of eight replicates. Left: upper row in (f). Right: lower row in (f)

GSNOR1 plays a critical role in governing protein‐SNO levels during plant immune responses and GSNOR1 activity controls the level of both GSNO and global protein‐SNOs (Chen et al., 2009; Feechan et al., 2005; Lee et al., 2008). First, the transcript level of *GSNOR1* on *P. parasitica* inoculation was determined. *GSNOR1* transcripts were significantly increased after 6 hr of infection by *P. parasitica*, but had decreased by 9 hpi (Figure 3a). This was accompanied by a concomitant increase in GSNOR1 activity following pathogen challenge (Figure 3b), suggesting the involvement of GSNOR1 in plant resistance to *P. parasitica*. To further elucidate the role of GSNOR1, loss‐of‐function mutants *gsnor1‐3* and *par2‐1* were challenged by *P. parasitica*. Water‐soaked lesions were produced as a result of *P. parasitica* inoculation in both *gsnor1‐3* and *par2‐1* detached leaves at 2 days postinoculation (dpi) (Figure S2a). The pathogen:host biomass ratio assay also showed significantly higher and faster proliferation of *P*. *parasitica* in *gsnor1‐3* and *par2‐1* plants as compared to wildtype Col‐0 plants (Figure 3c), indicating enhanced susceptibility of these plants to *P. parasitica* infection. Determination of pathogen colonization and spread of mycelium in Col‐0 and *gsnor1‐3* plants via trypan blue staining also showed significantly higher growth of *P. parasitica* in *gsnor1‐3* plants (Figure 3d,e). Similar results were observed in green fluorescent protein (GFP)‐expressing *P. parasitica*‐inoculated plants (Figure 3f,g). A *GSNOR* overexpression line, *gsnor1‐1*, was also scored for its response to *P. parasitica*: this line did not show a significant difference compared with wildtype Col‐0 plants (Figure S2b,c). Collectively, these results indicate that *GSNOR1* plays a key role in *Arabidopsis* basal resistance against *P. parasitica*.

*P. parasitica* is a typical soilborne pathogen and mainly infects the roots and crown area of the stem. We challenged in vitro grown seedlings with *P. parasitica* by inoculating them with culture plugs placed on the crown area. As expected, *gsnor1‐3* and *par2‐1* plants exhibited an enhanced death rate relative to Col‐0 at 4 dpi (Figure S2d). These results show that GSNOR1 plays a key function in basal resistance of *Arabidopsis* against *P. parasitica* challenge.

### 2.3 GSNOR1 is required for SA signalling during basal resistance against *P*. *parasitica*

**FIGURE 4:**
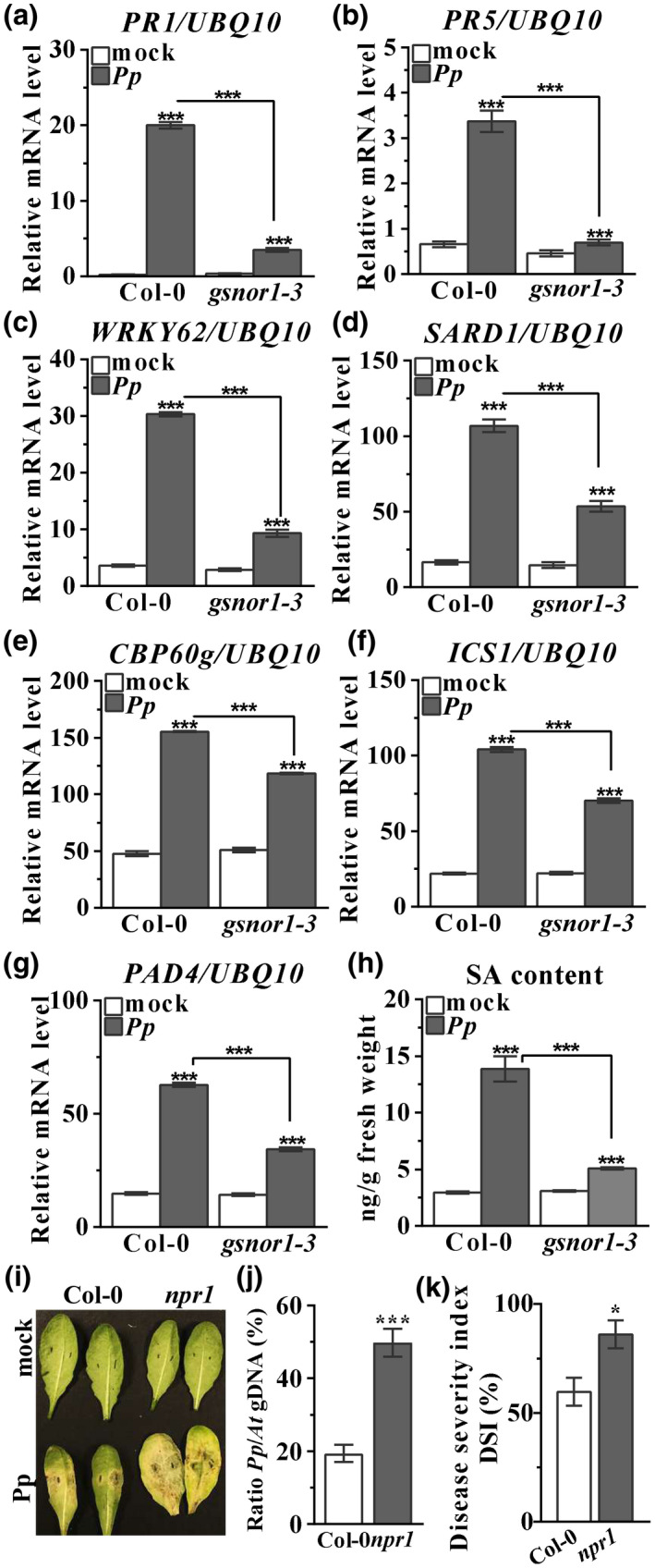
*GSNOR1* is required for salicylic acid (SA) synthesis and signalling during *Phytophthora* *parasitica* infection. (a–f) Expression of SA marker genes *PR‐1* (a), *PR5* (b), and *WRKY62* (c), and SA synthesis‐related genes *SARD1* (d), *CBP60g* (e), *ICS1* (f), and *PAD4* (g) were analysed. Ten‐day‐old wildtype (Col‐0) and *gsnor1‐3* seedlings were inoculated with *P. parasitica* or mock‐treated for 12 hr and then collected for quantitative reverse transcription PCR. Relative gene expression was normalized against the constitutively expressed gene, *UBQ10*. (h) Total SA levels in the leaves of wildtype (Col‐0) or *gsnor1‐3* plants 24 hr after *P. parasitica* inoculation. (i) Wildtype Col‐0 and *npr1* plants were infected with *P. parasitica* and disease symptoms recorded at 3 days postinoculation. (j) Pathogen biomass analysis by quantitative PCR for (i). (k) Incidence of *P parasitica* on wildtype Col‐0 and *npr1* seedlings on *P. parasitica* inoculation was analysed by disease severity index (DSI). The mean of the DSI was obtained from 16 seedlings, error bars represent *SD* from three replicates. Error bars indicate ± *SD* of three biological replicates. *p < .05, ***p < .001

A potential role for SA signalling in *Arabidopsis–P. parasitica* interactions has not been explored in detail to date. Thus, we examined SA‐related defence gene expression in *Arabidopsis* following attempted *P. parasitica* infection. As shown in Figure 4a–c, expression of the SA defence marker genes *PR1*, *PR5*, and *WRKY62* was significantly lower in the *gsnor1‐3* line relative to Col‐0 plants on *P. parasitica* infection (Figure 4a–c). These results indicate that SA signalling is engaged during basal disease resistance against *P. parasitica* and that this response is regulated by GSNOR1 function.

These findings prompted us to investigate if SA biosynthesis is associated with basal disease resistance in response to attempted *P. parasitica* infection. First, we measured the expression of *CBP60g*, *SARD1*, *ICS1*, and *PAD4*, which encode key SA biosynthesis and regulatory proteins, respectively (Zhang & Li, 2019). Interestingly, the induction of all these SA‐related genes following *P. parasitica* inoculation was significantly lower in *gsnor1‐3* plants (Figure 4d–g), implying that GSNOR1 might also regulate SA biosynthesis. As anticipated, the total SA content in *gsnor1‐3* plants was significantly lower than Col‐0 plants following *P. parasitica* inoculation (Figure 4h). Collectively, these data demonstrate that *GSNOR1* is a positive regulator of both SA accumulation and signalling during basal disease resistance against *P. parasitica*.

NPR1 is a key regulator of SA signalling and associated SA‐dependent gene expression (Cao et al., 1997). Leaves from 4‐week‐old Col‐0 and *npr1* plants were therefore subjected to *P. parasitica* inoculation. Phenotypic observations showed the appearance of severe symptoms on the leaves of *npr1* plants but not on those of Col‐0 plants, indicating enhanced disease susceptibility of *npr1* to *P. parasitica* (Figure 4i). The *npr1* line also supported significantly higher and faster proliferation of *P. parasitica* as determined by biomass analysis (Figure 4j). Furthermore, *npr1* plants also exhibited a higher level of disease severity index relative to Col‐0 plants (Figure 4k). Collectively, these data show that SA‐dependent responses are required for immunity against *P. parasitica* and GSNOR1 is required for both SA biosynthesis and signalling during attempted *P. parasitica* infection.

### 2.4 Loss of GSNOR1 function reduces *P. parasitica*‐triggered ROS production

**FIGURE 5:**
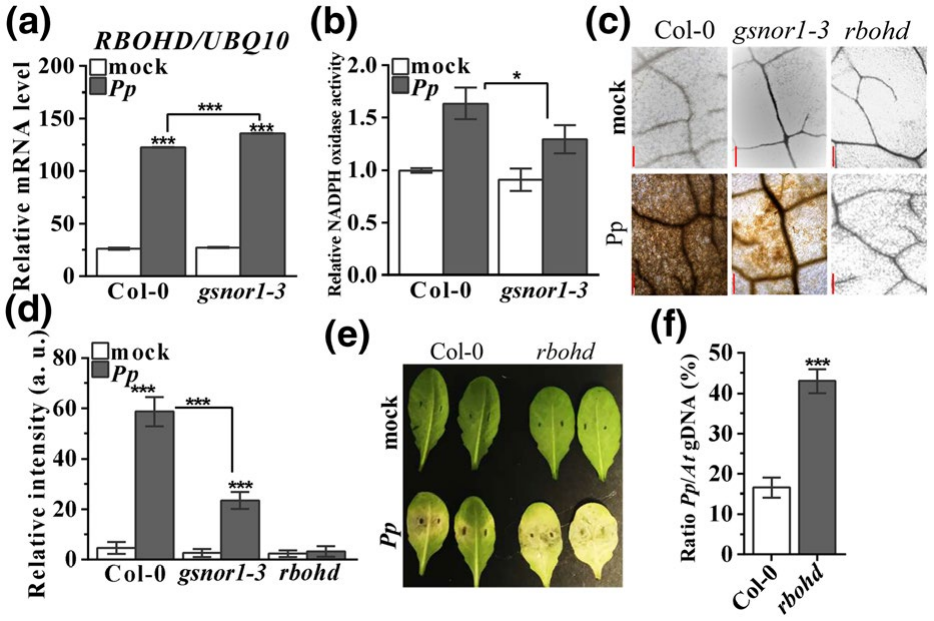
GSNOR1 function impacts reactive oxygen species (ROS) burst. (a) *RBOHD* transcripts were measured in wildtype (Col‐0) and *gsnor1‐3* plants after mock‐ or *Phytophthora parasitica* inoculation. Error bars represent *SD* of three biological replicates. (b) RBOHD activity analysed by relative NADPH oxidase activity in given *Arabidopsis* lines on challenge with V8 plug (mock) or *P. parasitica* (*Pp*). Error bars represent *SD* of three biological replicates. (c) Accumulation of hydrogen peroxide detected by diaminobenzidine (DAB) staining. Leaves were inoculated with a *P. parasitica* plug without any wounding, then stained with DAB solution. Images were taken at inoculation sites after destaining. Scale bars, 200 μm. (d) Quantification of DAB stain in (c) determined by ImageJ software. Error bars represent *SD* of eight replicates. (e) Wildtype Col‐0 and *rbohd* plants were infected with *P. parasitica* and disease symptoms were recorded at 3 days postinoculation. (f) Pathogen biomass analysis by quantitative PCR. Error bars indicate *SD* of three biological replicates

The oxidative burst is an early immune response and it has been reported that the timing of ROS production is an important determinant during the plant defence response to *P. parasitica* (Grant & Loake, 2000; Wi et al., 2012). Therefore, transcript accumulation of *Respiratory Burst Oxidase Homolog D* (*RBOHD*), which encodes a key enzyme for pathogen‐triggered ROS production (Torres et al., 2002), was analysed in response to *P. parasitica*. *RBOHD* was induced in wildtype plants following *P. parasitica* inoculation. Surprisingly, *RBOHD* expression in *gsnor1‐3* plants was higher compared to Col‐0 after infection with *P. parasitica* (Figure 5a). However, RBOHD activity was lower in the *gsnor1‐3* line compared to Col‐0 on infection (Figure 5b). Subsequently, we monitored ROS production in plants challenged with *P. parasitica*. Reduced diaminobenzidine (DAB) staining, a ROS‐sensitive marker (Thordal‐Christensen et al., 1997), was observed at *gsnor1‐3* plant inoculation sites (Figure 5c) relative to the wild type and quantification of DAB staining also showed significantly lower ROS accumulation in the *gsnor1‐3* mutant in response to *P. parasitica* infection (Figure 5d). To investigate the potential involvement of RBOHD, a major source of apoplastic ROS (Torres et al., 2002), in response to *P. parasitica* infection, we inoculated the *Arabidopsis rbohd* loss‐of‐function mutant (Torres et al., 2002) with *P. parasitica*. The *rbohd* line was highly susceptible to infection and exhibited enhanced disease symptoms as compared to wildtype plants (Figure 5e). Furthermore, relative biomass quantification of *P. parasitica* was undertaken via quantitative PCR (qPCR): enhanced levels of *P. parasitica* DNA was detected in *rbohd* plants relative to the wild type (Figure 5f), demonstrating the enhanced disease susceptibility of *rbohd* plants to *P. parasitica*.

### 2.5 GSNOR1 has a global impact on ROS and SA‐mediated gene expression

**FIGURE 6:**
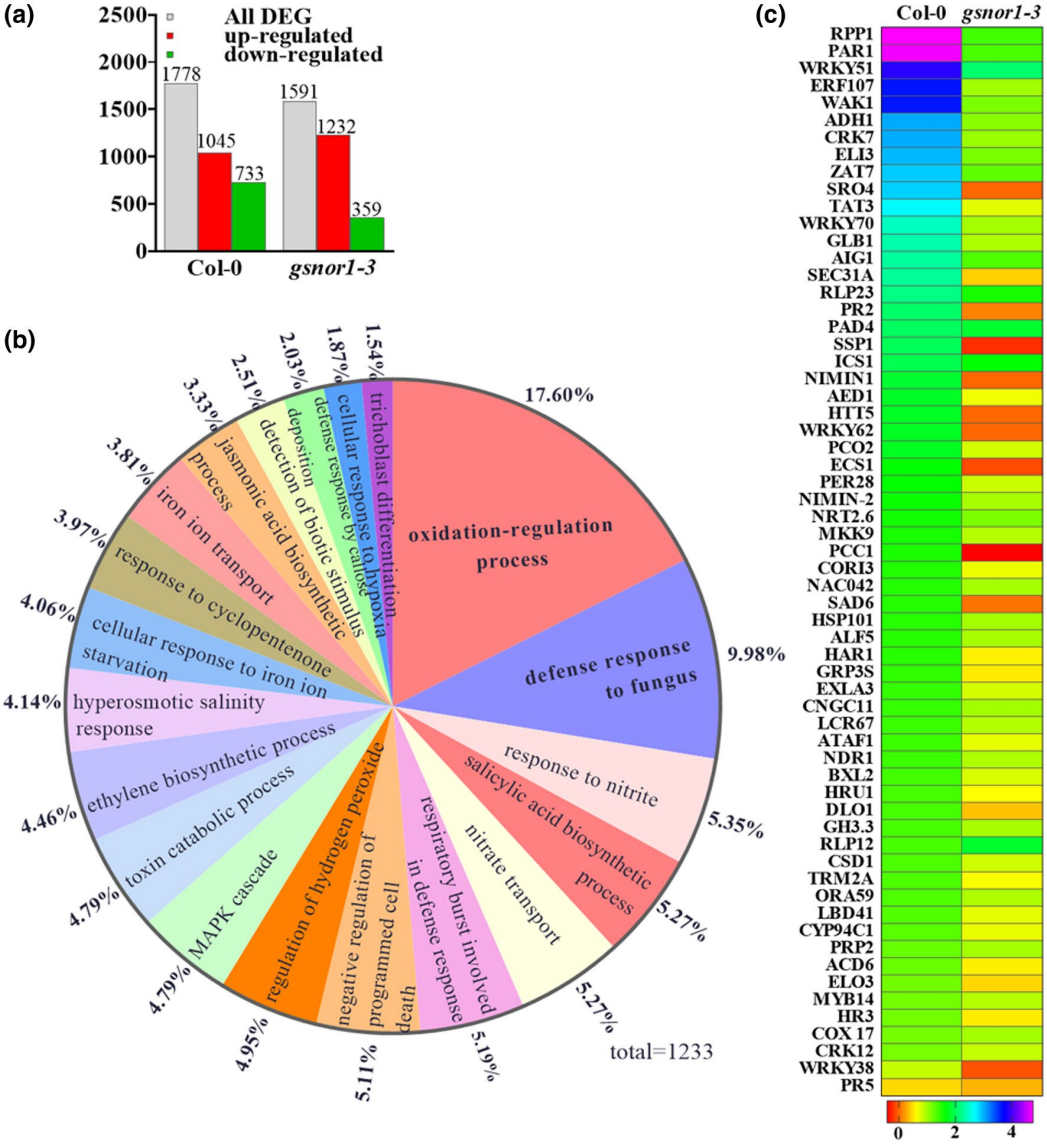
Transcriptional analysis of selected genes following *Phytophthora* *parasitica* infection. (a) The number of genes found to be upregulated or downregulated in wildtype Col‐0 and *gsnor1‐3* plants in response to *P. parasitica* infection compared to mock‐treated. (b) The 20 most enriched GO terms in biological process. There are 1,233 differentially expressed genes (DEGs) and the percentage of each GO term is indicated. (c) Heat map of the normalized RNA‐Seq data for genes involved in biotic stress response after inoculation with *P*. *parasitica*

To determine the impact of loss of *GSNOR1* function on global gene expression following *P. parasitica* inoculation, we performed RNA‐Seq‐mediated transcriptomic analysis of wildtype and *gsnor1‐3* seedlings. Significant changes in the expression of 1,778 differentially expressed genes (DEGs) were found in wildtype plants after 24 hr of infection, of which 1,045 were upregulated, whereas 733 were downregulated (Figure 6a). On the other hand, 1,591 DEGs were identified in *gsnor1‐3*, of which 1,232 were upregulated and 359 were downregulated (Figure 6a). This suggests that *GSNOR1* function is required for significant gene expression reprogramming during *P. parasitica* infection.

Gene ontology (GO) analysis identified enriched categories for DEGs related to biotic and abiotic stress responses (Figure 6b), indicating *GSNOR1* has a global impact on the *Arabidopsis* hormone, immune function, and oxidation‐associated gene expression profile. Specifically, we found that the oxidative stress response and response to hydrogen peroxide were more significantly affected in the *gsnor1‐3* line in response to *P. parasitica* as compared to wildtype plants (Figure 6c, Table 1). However, genes related to the oxidation–reduction process were more strongly impacted in wildtype plants relative to the *gsnor1‐3* line. These ROS‐related genes include *ALCOHOL DEHYDROGENASE 1, ADH1* (De la Rosa et al., 2019) and zinc finger protein 7, *ZAT7* (Ciftci‐Yilmaz et al., 2007). This suggests that *GSNOR1* is a key regulator of ROS regulation in response to *P. parasitica*. This again highlights the importance of ROS regulation by GSNOR1 in response to *P. parasitica* infection.

**TABLE 1:** Biological process gene ontology (GO) terms involved in oxidative responses and immune function are significantly different in *gsnor1‐3* mutants compared with the wild type on *Phytophthora* *parasitica* infection compared with mock

GO accession no.	Description	*p* value		Fold change
		Col‐0	*gsnor1‐3*	
GO:0009617	Response to bacterium	4.33E−06	1.20E−03	277.14
GO:0046244	Salicylic acid catabolic process	5.75E−03	1.00E+00	173.91
GO:0009751	Response to salicylic acid	4.28E−06	1.97E−04	46.03
GO:0009816	Defence response to bacterium	2.04E−05	3.38E−04	16.57
GO:0070301	Cellular response to hydrogen peroxide	4.54E−04	3.33E−03	7.33
GO:0010200	Response to chitin	1.00E−12	3.00E−12	3.00
GO:0009873	Ethylene‐activated signalling pathway	1.00E−12	2.00E−12	2.00
GO:0009862	Systemic acquired resistance, salicylic acid‐mediated signalling pathway	1.00E−12	2.00E−12	2.00
GO:2000031	Regulation of salicylic acid‐mediated signalling pathway	4.55E−01	1.00E+00	2.20
GO:0042742	Defence response to bacterium	3.00E−12	4.00E−12	1.33
GO:0045454	Cell redox homeostasis	5.77E−01	6.83E−01	1.18
GO:0009867	Jasmonic acid‐mediated signalling pathway	1.00E−12	1.00E−12	1.00
GO:0009753	Response to jasmonic acid	1.00E−12	1.00E−12	1.00
GO:0034599	Cellular response to oxidative stress	7.98E−01	7.38E−01	0.92
GO:0071395	Cellular response to jasmonic acid stimulus	3.11E−02	2.58E−02	0.83
GO:0080142	Regulation of salicylic acid biosynthetic process	1.64E−03	1.23E−03	0.75
GO:0009611	Response to wounding	4.00E−12	2.00E−12	0.50
GO:0010363	Regulation of plant‐type hypersensitive response	4.00E−12	2.00E−12	0.50
GO:0009620	Response to fungus	5.00E−12	2.00E−12	0.40
GO:0071323	Cellular response to chitin	4.93E−02	2.91E−03	0.06

Furthermore, we found that genes integral to SA‐related signalling were strongly differentially expressed in wildtype plants but not in the *gsnor1‐3* line in response to *P. parasitica* inoculation (Table 1). This data suggests that SA signalling plays an important role in resistance against *P. parasitica* and GSNOR1 is a key regulator of these responses. Interestingly, jasmonic acid (JA) signalling seems not to be regulated by *GSNOR1* on *P. parasitica* inoculation. Collectively, these results suggest that *GSNOR1* has significant impact on SA signalling in response to attempted *P. parasitica* infection.

### 2.6 Secreted proteins from *P. parasitica* inhibit GSNOR1 activity

**FIGURE 7:**
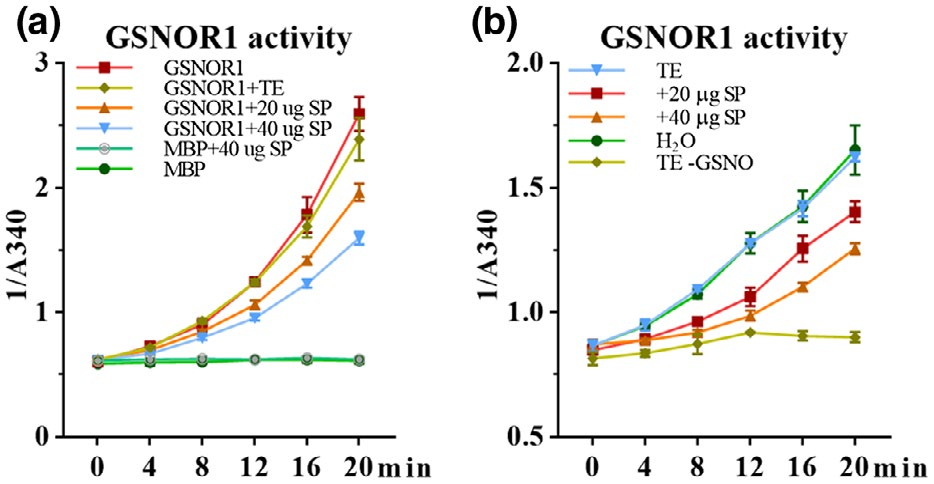
Secreted proteins from *Phytophthora* *parasitica* suppress GSNOR1 activity. (a) Secreted proteins (SP) from *P. parasitica* inhibit MBP‐GSNOR activity. Maltose binding protein (MBP) and MBP‐GSNOR1 were expressed in vitro and used for GSNOR activity assays. Secreted proteins of *P. parasitica* were extracted and resolved in TE buffer. TE buffer was treated as mock. (b) *Arabidopsis* GSNOR activity assay. Total protein from secreted protein or TE‐pretreated *Arabidopsis* seedlings were extracted and used for GSNOR activity assays. TE−GSNO was used as a negative control (without GSNO)

As GSNOR1 function is important for basal resistance against *P. parasitica*, we analysed whether the secreted proteome of *P. parasitica* might target GSNOR1 activity. Freshly secreted total protein from *P. parasitica* was collected as previously described (Kamoun et al., 1993). Recombinant GSNOR1 was incubated with the *P. parasitica* secreted proteome. Interestingly, the secreted proteome of *P. parasitica* exhibited a dose‐dependent inhibition of GSNOR activity (Figure 7a). Furthermore, treatment of *Arabidopsis* seedlings with a *P. parasitica* secreted proteome extract inhibited plant GSNOR activity (Figure 7b).

**FIGURE 8:**
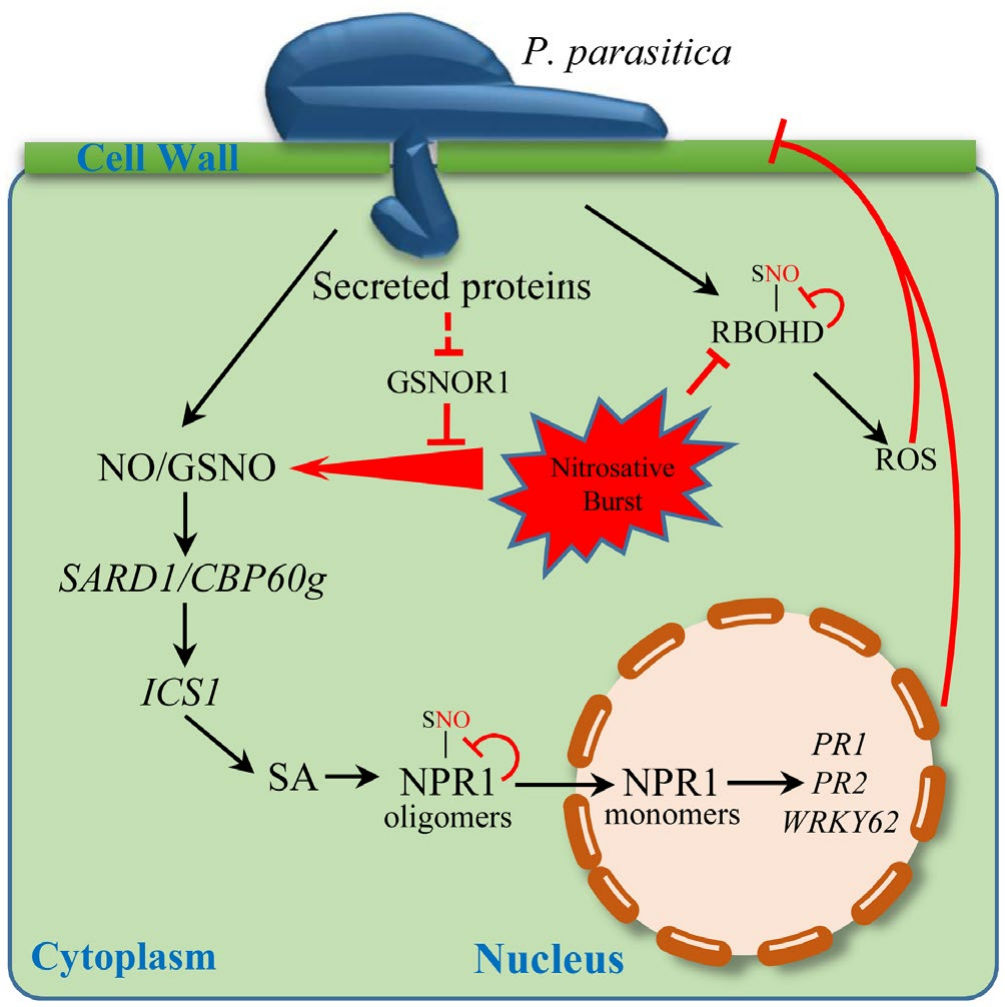
Schematic model showing the roles of NO and GSNOR1 in the *Arabidopsis–Phytophthora* *parasitica* interaction. Proposed biological function of NO and GSNOR1 in the *Arabidopsis–P. parasitica* interaction. Blunt arrows indicate negative regulation and pointed arrows represents positive regulation. *P. parasitica* infection induces NO and subsequently GSNO, which activate defence responses including salicylic acid (SA) signalling, leading to the restriction of *P. parasitica* infection. However, as NO and GSNO concentrations increase, RBOHD, the major leaf NADPH oxidase, is *S*‐nitrosylated at Cys^890^, decreasing its activity (Yun et al., 2011). In parallel, NPR1 is *S*‐nitrosylated, promoting its cytosolic localization and blunting SA‐dependent gene expression (Tada et al., 2008). Collectively, these molecular events in combination may curb the immune response. During infection *P*. *parasitica* might deliver secreted proteins into host cells to target GSNOR1 leading to excessive (S)NO accumulation to promote increased susceptibility

Based on our data, we propose a model for the role of *GSNOR1* in basal disease resistance against *P. parasitica* (Figure 8). Attempted *P. parasitica* infection induces NO and subsequently GSNO accumulation, which activate defence responses, leading to a restriction of *P. parasitica* infection. In addition, RBOHD is activated, enhancing ROS production, resulting in inhibition of *P. parasitica* infection. However, when NO and GSNO accumulate to relatively high levels, excessive *S*‐nitrosylation might result in defence suppression and associated pathogen susceptibility.

## 3 DISCUSSION

Our findings show that the level of NO is crucial for establishing basal resistance against *P. parasitica* in *Arabidopsis*. Thus, counterintuitively either a relatively low or relatively high level of NO accumulation leads to reduced basal resistance against *P. parasitica*. Thus, perturbations in NO homeostasis promote *Arabidopsis* disease susceptibility towards *P. parasitica*. Furthermore, loss‐of‐function mutations in *GSNOR1* disable both SA accumulation and signalling, and also ROS accumulation, leading to enhanced susceptibility towards *P. parasitica*. Significantly, we have demonstrated that an extract of the secreted proteome from *P. parasitica* inhibits GSNOR1 activity. Because GSNOR1 activity is required for SA signalling and ROS production, *P. parasitica* may target GSNOR1 to aid pathogenesis, facilitating colonization.

Based on our data, we propose a model for the role of *GSNOR1* in basal disease resistance against *P. parasitica* (Figure 8). We have shown that attempted *P. parasitica* infection induces NO and subsequently GSNO accumulation. Subsequently, these molecules activate defence responses, such as SA signalling, leading to a restriction of *P. parasitica* infection. In addition, RBOHD is activated, enhancing ROS production, resulting in inhibition of *P. parasitica* infection. However, as NO and GSNO concentrations increase, their homeostasis is perturbed, and excessive *S*‐nitrosylation might result in defence suppression and associated pathogen susceptibility. For example, increasing NPR1 *S*‐nitrosylation sequesters this transcriptional co‐activator in the cytosol, blunting SA‐dependent gene expression (Tada et al., 2008). Also, *S*‐nitrosylation of SA‐binding protein 3 (SABP3) reduces the SA binding of this protein and also the carbonic anhydrase activity of this enzyme, increasing disease susceptibility (Wang et al., 2009). In parallel, the nicotinamide adenine dinucleotide phosphate (NADPH) oxidase, RBOHD, is *S*‐nitrosylated at Cys890, decreasing its activity (Yun et al., 2011) and suppressing ROS production. Collectively, these molecular events in combination may curb the immune response. Thus, perturbations in NO/GSNO homeostasis may promote increased susceptibility towards *P. parasitica*.

### 3.1 NO homeostasis is a critical component of plant resistance against *P*. *parasitica*

Previous studies have revealed that NO is induced during the establishment of resistance against *P. capsici* challenge (Requena et al., 2005) or by defence‐activating elicitors from *Phytophthora cryptogea* (Foissner et al., 2000). Our data extend these findings by suggesting that *P. parasitica* infection (in addition to resistance) also triggered a NO burst in *Arabidopsis*. Application of the NO donor SNP has been reported to enhance potato susceptibility to *P. infestans* infection (El‐Beltagi et al., 2017). In contrast, we found that application of either NO scavengers or nitric oxide synthase (Ichinose et al., 2003) inhibitors blunted *Arabidopsis* resistance to *P. parasitica*. Also, the NR‐deficient mutant *nia1 nia2* displayed reduced *P. parasitica*‐induced NO production and enhanced susceptibility to *P. parasitica*. This is consistent with previous data that suggests that NR‐dependent NO production is required for resistance to *P. infestans* (Floryszak‐Wieczorek et al., 2016).

The NO overproduction mutant, *nox1*, also showed increased susceptibility to *P. parasitica* relative to the wild type (Figure 1). Furthermore, loss of *GSNOR1* function in the *gsnor1‐3* mutant, which elevates both NO and GSNO, also showed enhanced susceptibility to *P. parasitica* (Figure 2), suggesting excessive endogenous NO and/or GSNO negative feedback regulates immunity against *P. parasitica*. In this context, it has been shown that endogenous NO and GSNO, two pivotal redox signalling molecules, may have both distinct and overlapping functions during the development of immunity (Yun et al., 2016). Collectively, our data imply there is a breadth of NO concentration that supports the establishment of optimal basal resistance against *P. parasitica*. Hence, by extension, if the level of NO falls outside this concentration range then basal resistance against *P. parasitica* is reduced.

### 3.2 GSNOR1 is required for basal resistance against *P*. *parasitica*

The mutant lines *gsnor1‐3* and *par2‐1*, another allele of *GSNOR1* (Chen et al., 2009) that exhibits a high level of SNO, both showed enhanced susceptibility to *P. parasitica*. Consistent with our study, previous research also found that the absence of GSNOR1 activity might be involved in potato susceptibility to *P. infestans* (Abramowski et al., 2015). Interestingly, we also found that the NO overproduction mutant *nox1* also showed enhanced susceptibility to *P*. *parasitica*. It has been shown that GSNOR1 is *S*‐nitrosylated, inhibiting its activity (Frungillo et al., 2014). Thus, high NO levels in this mutant could inhibit GSNOR1 activity through *S*‐nitrosylation, leading to excessive (S)NO levels. Consistently, GSNOR activity is required for plant basal immunity (Feechan et al., 2005; Yun et al., 2016). Furthermore, GSNOR1 activity has been shown to be differently modulated in lettuce against downy mildew (Tichá et al., 2018; Yun et al., 2016). Collectively, our data establish that GSNOR1 function is required for *Arabidopsis* resistance against *Phytophthora*.

### 3.3 NO‐mediated regulation of SA signalling integral for *P. parasitica* resistance

The immune regulator SA plays a central role in the plant immune response, with SA biosynthesis and signalling under redox regulation (Feechan et al., 2005; Lindermayr et al., 2010; Tada et al., 2008). Although SA function has been shown to be important for immunity against a number of diverse plant pathogens (Liu et al., 2014; Pan et al., 2016; Yang et al., 2017), a potential role for SA in resistance against *P. parasitica* has not been thoroughly investigated. We have previously reported that the *Arabidopsis* mutant, resistance to *P. parasitica1* (*rtp1*), engaged SA signalling and exhibited resistance to *P. parasitica* (Pan et al., 2016). Recently, the *Arabidopsis rtp5* line has also been shown to be associated with increased SA synthesis and enhanced protection against *P. parasitica* infection (Li et al., 2020). Here we show SA‐mediated transcriptional reprogramming is impaired in *gsnor1‐3* plants in response to *P. parasitica*. The SA‐signalling mutant *npr1* also showed susceptibility to *P. parasitica*. Furthermore, in *gsnor1‐3* plants, increased SNO levels have been shown to compromise SA signalling by both inhibiting SABP3 function and controlling the nuclear translocation of NPR1 (Tada et al., 2008; Wang et al., 2009). Thus, GSNOR1 may regulate SA‐dependent basal resistance against *P. parasitica* in a similar fashion.

### 3.4 *P. parasitica‐*induced NO regulates the ROS burst

The timing of ROS production is an important determinant in plant compatible interactions towards *P. parasitica* (Pan et al., 2016; Wi et al., 2012). NADPH oxidases have been uncovered as an essential enzyme for ROS production in plant defence and previous reports have shown that attempted *Phytophthora* infection could induced *RBOHD* expression and enhance the ROS burst. Moreover, it has been shown that resistance in the root of *Arabidopsis* against *P. parasitica* requires an NADPH oxidase‐mediated oxidative burst: NADPH oxidase knockdown or knockout plants showed increased susceptibility to oomycetes (Shibata et al., 2010). Recently, the effector RxLR207 from *P. capsici* was shown to target BPA1 (Binding partner of ACD11), which may control ROS production (Li et al., 2019). Also, we reported a T‐DNA insertion mutant, *rtp1‐1*, that disables a nodulin‐related MtN21 family protein, resulting in increased ROS production and enhanced resistance to *P. parasitica* (Pan et al., 2016). Our findings here suggest that *rbohd* mutants, disabled in the most important NADPH oxidase isoform associated with the leaf oxidative burst (Torres et al., 2002), showed increased susceptibility to *P. parasitica*. Furthermore, the oxidative burst was reduced in *gsnor1‐3* plants relative to the wild type, suggesting that NADPH oxidase function is downregulated in this line. However, even though *gsnor1‐3* plants are defective in SA signalling, transcripts of *RBOHD* were induced in a similar fashion in both wildtype and *gsnor1‐3* plants. Therefore, RBOHD function is likely to be regulated in a posttranscriptional or posttranslational fashion. Indeed, during the immune response to attempted *Pseudomonas syringae* pv*. tomato* DC3000 (*avrB*) infection, RBOHD activity is regulated by *S*‐nitrosylation of Cys890 and this redox‐based control mechanism is conserved in both flies and humans (Yun et al., 2011).

### 3.5 *P. parasitica*‐secreted proteins target GSNOR1 activity

Oomycete pathogens secrete a series of proteins that interfere with plant immune function by targeting key regulators of plant immunity (Jiang & Tyler, 2012; Wang et al., 2019). Interestingly, it has recently been reported that host plants can disarm the bacterial effector HopAI1 by *S*‐nitrosylation, demonstrating a function for NO production in the neutralization of pathogen effectors (Ling et al., 2017). Furthermore, it has been reported that *Phytophthora* effectors can interfere with the host plant immune system. For example, *Phytophthora* effector PsCRN63 can target CAT2 to regulate ROS homeostasis (Li et al., 2016). RxLR48, an effector from *P. capsici*, has been reported to target NPR1, triggering its proteasome‐mediated degradation (Li et al., 2019). Our data suggests that one or more secreted proteins from *P. parasitica* might inhibit GSNOR1 activity. Thus, our study reveals a potential novel molecular mechanism where secreted *P. parasitica* proteins may target GSNOR1 for inactivation to promote pathogenesis.

In conclusion, we provided genetic evidence that NO and GSNOR1 are required for *Arabidopsis* basal resistance against *P. parasitica*. Furthermore, our study suggests that NO‐mediated *S*‐nitrosylation regulates both SA signalling and the ROS burst against *P. parasitica*. We also provide preliminary evidence that one or more secreted proteins from *P. parasitica* can inhibit GSNOR1 activity. Future work will endeavour to uncover the identity of protein(s) that target GSNOR1 and the underpinning mechanism that promotes the inhibition of this key immunity‐related enzyme. This work may provide new avenues for molecular breeding strategies to convey improved resistance in crop plants against oomycete infection.

## 4 EXPERIMENTAL PROCEDURES

### 4.1 Plant materials and growth conditions

The *A*. *thaliana* wildtype accession Columbia‐0 (Col‐0), Landsberg *erecta* (Ler), Wassilewskija (Ws), and the mutant lines *gsnor1‐3*, *par2‐1* (Chen et al., 2009; Feechan et al., 2005), *nox1* (Li et al., 1995), *rbohd* (Torres et al., 2002), *nia1 nia2* (Wilkinson & Crawford, 1993), and *npr1* (Cao et al., 1997) were used in this research.

Seeds were surface‐sterilized by 70% (vol/vol) ethanol and antiformin, then planted on to 1/2 × Murashige and Skoog (MS) plates (Wang et al., 2011). Five‐day‐old seedlings were used for 4‐amino‐5‐methylamino‐2′,7′‐difluorofluorescein (DAF‐FM; Sigma) staining and 10‐day‐old plants were used for seedling inoculation. For other experiments, 10‐day‐old seedlings were transferred to soil and grown under short‐day conditions (10 hr light/14 hr dark). Four‐week‐old plants were used for detached leaf inoculation.

### 4.2 Pathogen infection assays

The *P*. *parasitica* strain Pp016 used in this study was cultured at 25 °C in the dark on 10% (vol/vol) V8 agar medium (10% V8 juice, 0.1% CaCO_3_, 2% agar). Mycelial plugs of 1 cm^2^ size were cut and grown on fresh V8 juice medium (10% V8 juice, 0.1% CaCO_3_) for 3 days. Production of zoospores was initiated by cold and salt treatment (Wang et al., 2011). Zoospores were collected and the concentration was adjusted to 10^5^ zoospores/ml for all experiments unless otherwise specified.

For the inoculation of detached leaves, 10 leaves of each line were drop‐inoculated with zoospores or sterile distilled water. The leaves were observed following the biomass assay. For colonized agar plug inoculation, Pp016 (cultured in fresh V8 medium for 5 days) and the V8 agar (control) were used. For this purpose, plugs of 5 mm diameter or width were cut under sterile conditions and placed on the root of 12‐day‐old seedlings. Disease development was evaluated using a disease severity index (DSI) on a scale of 0–3, in which 0 means no visible disease symptoms, 1 indicates one or two leaves collapsed, 2 indicates three or four leaves collapsed, and 3 indicates more than five leaves collapsed; seedlings with the shoot apex collapsed were scored as 3 directly. Sixteen plants were used in each assay, and the experiments were repeated three times. DSI (%) = [sum (the number of seedlings in this index × disease index)]/48 × 100.

### 4.3 NO measurement

The endogenous levels of NO in *Arabidopsis* roots were determined by DAF‐FM diacetate staining under TCS SP5 fluorescence microscopy (Leica). Briefly, 5‐day‐old seedlings were inoculated with Pp016 zoospores (5 × 10^5^spores/ml). Samples were collected after 0, 3, 6, and 9 hr and dipped to 10 µM DAF‐FM staining solution. For enhanced specificity, fluorescence related to Pp016‐induced NO was further confirmed by the application of 200 µM of the NO‐scavenger 2‐4‐carboxyphenyl‐4,4,5,5‐tetramethylimidazoline‐1‐oxyl‐3‐oxide (cPTIO; Sigma). Fluorescence was quantified by ImageJ software (Yun et al., 2011).

### 4.4 Biotin switch for total SNO measurement

*Arabidopsis* seedlings were inoculated with Pp016 zoospores and whole seedlings were collected after 0, 3, 6, and 9 hr. The sample was homogenized in HEN buffer (25 mM HEPES, pH 7.7, 1 mM EDTA, 0.1 mM neocuproine) containing 1× complete protease inhibitor cocktail (Roche) and centrifuged at 4 °C for 20 min at 10,000 × g. Total protein concentration in the supernatant was measured using the Bradford assay. About 200 µg of total protein from each sample was subjected to the biotin‐switch assay (Jaffrey & Snyder, 2001) and the resulting samples were labelled with 50 mM sodium ascorbate and 1 mM biotin‐HPDP or 50 mM sodium chloride and 1 mM biotin‐HPDP, which served as a negative control. *S*‐nitrosylated proteins were subjected to immunoblot analysis using an anti‐biotin antibody (anti‐biotin, horseradish peroxidase [HRP]‐linked antibody #7075; Cell Signaling Technology). Total protein input was visualized by immunoblot with anti‐actin antibody (60008‐1g; Proteintech) and mouse secondary antibody (anti‐mouse IgG, HRP‐linked antibody #7076; Cell Signaling Technologies).

### 4.5 GSNOR activity assay

GSNOR activity of total protein from *Arabidopsis* lines was measured at 25 °C by decomposition of NADH at 340 nm (Feechan et al., 2005). Samples were collected at indicated times and associated activity was determined by incubating 100 µg of plant extracted total protein in 1 ml of reaction mix containing 20 mM Tris‐HCl (pH 8.0), 0.2 mM NADH, and 0.5 mM EDTA. The reaction was initiated by the addition of GSNO to the mix at a final concentration of 300 µM. The resulting GSNOR activity was expressed as nmol NADH degraded min^−1^⋅mg^−1^ protein.

To analyse the effect of secreted proteins from Pp016 on the activity of recombinant maltose binding protein (MBP) or MBP‐GSNOR1 protein, the purified recombinant protein was desalted with Zeba Spin column (Thermo Fisher Scientific). Then, 1 mM MBP or MBP‐GSNOR1 was subjected to GSNOR activity measurements in the presence of 20 or 40 µg of secreted protein from Pp016 and measured every 4 min for 20 min. The resulting GSNOR activity was expressed as 1/A_340_ to represent GSNOR‐dependent NADH consumption.

### 4.6 RBOHD activity assay

RBOHD activity was measured as NADPH oxidase activity as described previously (Yun et al., 2011). Briefly, the membrane fraction from 1 g of *Arabidopsis* leaf was ground and extracted in extraction buffer (0.25 M sucrose, 50 mM HEPES, pH 7.2, 3 mM EDTA, 1 mM dithiothreitol, 0.6% polyvinylpyrrolidone, 3.6 mM l‐cysteine, 0.1 mM MgCl_2_ including protease inhibitor tablet [Roche]) by ultracentrifugation. The membrane fraction was used to analyse NADPH oxidase activity using epinephrine and NADPH as substrates. The reaction was started with the addition of NADPH and the absorbance was measured at 480 nm by spectrophotometer.

### 4.7 RNA extraction and cDNA synthesis

RNA extraction was performed with a Plant RNA Isolation Mini Kit (Agilent Technologies). Briefly, about 100 mg plant leaves were collected in liquid N_2_ and homogenized with the tissue lyser (Qiagen) before resuspending in 500 μl of extraction buffer. The homogenate was transferred to a prefiltration column and spun down for 2 min at 16,000 × g before adding 500 μl of 2‐propanol to the flow‐through. The mixture was centrifuged through a mini‐isolation column and washed twice with 600 μl of wash buffer at 16,000 × g for 1 min. Finally, RNA was eluted with RNase‐free water and quantified with a NanoDrop 2000 spectrophotometer (Thermo Fisher Scientific). cDNA was synthesized from about 2 μg of total RNA using oligo(dT) primers and reverse transcriptase (First‐Strand cDNA Synthesis Kit; Invitrogen).

### 4.8 Quantitative PCR

To check the pathogen colonization levels a quantitative real‐time PCR analysis was conducted to compare *P. parasitica* UBC (*PpUBC*) DNA levels (to measure the pathogen biomass) to *Arabidopsis* UBC (*AtUBC9*) DNA levels (Pan et al., 2016; van Esse et al., 2008).

qPCR analysis for biomass assay and for checking the expression of all other genes was performed as described below, using the primers given in Table S1. qPCR was performed using the LightCycler 480 Real‐Time PCR System (Roche). Gene expression levels were quantified by LightCycler DNA Master SYBR Green I mix and LightCycler system and gene expression values were determined using *UBQ10* as reference. All experiments were repeated at least three times.

### 4.9 DAB and trypan blue staining

To quantify the various ROS species in the *Arabidopsis* lines before and after pathogen inoculation, leaf samples were submerged in 0.5 mg/ml nitroblue tetrazolium (DAB; Sigma) staining buffer for 3 hr at room temperature in the dark. Leaves were then destained in 70% ethanol until the green colour was completely removed. Leaves were observed with a microscope (Olympus) and photographed.

To observe the proliferation of *P. parasitica* hyphae during infection, uninoculated control and inoculated *Arabidopsis* leaves were stained in trypan blue solution (10 g phenol, 10 ml glycerol, 10 ml lactic acid, 10 ml water, 10 mg trypan blue). For this purpose, leaves were dipped in the staining solution inside small glass bottles submerged in boiling water for 2 min. After cooling to room temperature, the samples were destained with 2.5 g/ml chloral hydrate solution until the samples were clean of any residual stain. The samples were rinsed with water and viewed under a microscope (Olympus).

### 4.10 Purification of total secreted protein from *P. parasitica* Pp016

*P. parasitica* strain Pp016 was grown on V8 agar for 5 days. Then small discs were transferred to 500 ml of synthesis liquid medium and total secreted proteins were collected as described (Kamoun et al., 1993). The total precipitated protein was dissolved in 2 ml of TE buffer and the protein concentration was quantified with the Bradford assay.

### 4.11 Recombinant protein expression and purification

For recombinant protein expression, *Arabidopsis GSNOR1* gene was cloned into MBP‐tagged expression vector pMAL‐c5×. Then, the vector pMAL‐c5× and pMAL‐c5×‐*GSNOR1* were transformed into *Escherichia coli* BL21(DE3) for MBP and MBP‐GSNOR1 expression, respectively. For recombinant protein production, overnight cultures grown at 37 °C were diluted 100‐fold in LB medium containing 100 µg/ml ampicillin and incubated until OD_600_ = 0.5. Then, 0.5 mM isopropyl β‐D‐1‐thiogalactopyranoside (IPTG; final concentration) was added and the cultures were incubated at 18 °C for 18 hr. The resulting bacterial cultures were collected by centrifuge and washed once by precooled phosphate‐buffered saline. Finally, recombinant protein was purified using amylose magnetic beads (New England Biolabs) under native conditions according to the manufacturer's instructions.

### 4.12 RNA sequencing and GO enrichment analysis

For transcriptome analysis, 10‐day‐old seedlings of Col‐0 and *gsnor1‐3* mutant were inoculated by *P. parasitica* or water and collected in liquid N_2_ at 12 hpi. Total RNA was extracted as described followed. Briefly, about 100 mg of sample homogenized in liquid N_2_ was added to 1 ml TRIzol reagent (Invitrogen) and the homogenate separated by centrifugation, followed by mixing the upper aqueous layer with chloroform. After centrifugation the aqueous layer was mixed with ethanol and added to RNase‐Free Columns (CR3) followed by two washes with buffer RW1 and one wash with RW before RNA was resolved in RNase‐free water as described by the RNAprep Pure Plant Plus Kit instruction (Tiangen). A total 3 µg of high‐quality RNA per sample was used for sequencing on an Illumina NovaSeq 6000 platform and 150 bp paired‐end reads were generated. Reference genome and gene model annotation files were downloaded from the genome website directly (ftp://ftp.arabidopsis.org/home/tair) using TopHat v. 2.0.12. Cuffquant and cuffnorm v. 2.2.1 were used to calculate the fragments per kilobase of transcript per million mapped reads values of genes in each sample. RNA‐Seq data have been deposited in the ArrayExpress database at EMBL‐EBI (www.ebi.ac.uk/arrayexpress) under accession number E‐MTAB‐8845.

DESeq was used to identify DEGs with fold change ≥2 and false discovery rate <0.01. GO enrichment analysis of DEGs was performed by the GOseq R package, in which gene length bias was corrected. GO terms with corrected *p* value < .05 were considered significantly enriched. KOBAS v. 2.0 software was used for the GO term enrichment analysis.

## AUTHOR CONTRIBUTIONS

G.L. and Q.P. conceived and designed the experiments. B.C., X.M., Y.L., Y.Z., X.J., A.T., and B.Y. performed the experiments. B.C., X.M., Y.L., B.Y., J.L., and A.H. analysed the data. G.L. and Q.P. wrote the manuscript with contributions from all authors. All authors reviewed the manuscript. There is no conflict of interest for any coauthors.

## Supporting information

**FIGURE S1** Reduced NO levels affect *Arabidopsis* resistance to *Phytophthora parasitica*

**FIGURE S2***GSNOR1* loss‐of‐function mutants are more susceptible to *Phytophthora parasitica* infection

**FIGURE S3** Protein stability assay after GSNOR activity assay

**TABLE S1** Primer list

## Data Availability

The data that support the findings of this study are available from the corresponding author on reasonable request.

## References

[mpp13102-bib-0001] Abramowski, D., Arasimowicz‐Jelonek, M., Izbiańska, K., Billert, H. & Floryszak‐Wieczorek, J. (2015) Nitric oxide modulates redox‐mediated defense in potato challenged with *Phytophthora infestans* . European Journal of Plant Pathology, 143, 237–260.

[mpp13102-bib-0002] Astier, J., Rasul, S., Koen, E., Manzoor, H., Besson‐Bard, A., Lamotte, O. et al. (2011) *S*‐nitrosylation: an emerging post‐translational protein modification in plants. Plant Science, 181, 527–533. 21893248 10.1016/j.plantsci.2011.02.011

[mpp13102-bib-0003] Belhaj, K., Lin, B. & Mauch, F. (2009) The chloroplast protein RPH1 plays a role in the immune response of *Arabidopsis* to *Phytophthora brassicae* . The Plant Journal, 58, 287–298. 19170932 10.1111/j.1365-313X.2008.03779.x

[mpp13102-bib-0004] Cao, H., Glazebrook, J., Clarke, J.D., Volko, S. & Dong, X. (1997) The Arabidopsis *NPR1* gene that controls systemic acquired resistance encodes a novel protein containing ankyrin repeats. Cell, 88, 57–63. 9019406 10.1016/s0092-8674(00)81858-9

[mpp13102-bib-0005] Chen, R., Sun, S., Wang, C., Li, Y., Liang, Y., An, F. et al. (2009) The *Arabidopsis PARAQUAT RESISTANT2* gene encodes an *S*‐nitrosoglutathione reductase that is a key regulator of cell death. Cell Research, 19, 1377. 19806166 10.1038/cr.2009.117

[mpp13102-bib-0006] Ciftci‐Yilmaz, S., Morsy, M.R., Song, L., Coutu, A., Krizek, B.A., Lewis, M.W. et al. (2007) The EAR‐motif of the Cys2/His2‐type zinc finger protein Zat7 plays a key role in the defense response of *Arabidopsis* to salinity stress. Journal of Biological Chemistry, 282, 9260–9268. 10.1074/jbc.M611093200 17259181

[mpp13102-bib-0008] De la Rosa, C. , Covarrubias, A.A. & Reyes, J.L. (2019) A dicistronic precursor encoding miR398 and the legume‐specific miR2119 coregulates *CSD1* and *ADH1* mRNAs in response to water deficit. Plant, Cell & Environment, 42, 133–144. 10.1111/pce.13209 29626361

[mpp13102-bib-0009] Delledonne, M., Xia, Y., Dixon, R.A. & Lamb, C. (1998) Nitric oxide functions as a signal in plant disease resistance. Nature, 394, 585. 9707120 10.1038/29087

[mpp13102-bib-0010] Dodds, P.N. & Rathjen, J.P. (2010) Plant immunity: towards an integrated view of plant–pathogen interactions. Nature Reviews Genetics, 11, 539. 10.1038/nrg2812 20585331

[mpp13102-bib-0011] El‐Beltagi, H.S., Ahmed, O.K. & Shehab, G.M. (2017) Nitric oxide treatment and induced genes role against *Phytophthora infestans* in potato. Gesunde Pflanzen, 69, 171–183.

[mpp13102-bib-0053] van Esse, H.P. , Van't Klooster, J.W., Bolton, M.D., Yadeta, K.A., van Baarlen, P. , Boeren, S. et al. (2008) The *Cladosporium fulvum* virulence protein Avr2 inhibits host proteases required for basal defense. The Plant Cell, 20, 1948–1963. 18660430 10.1105/tpc.108.059394 PMC2518240

[mpp13102-bib-0012] Feechan, A., Kwon, E., Yun, B.‐W., Wang, Y., Pallas, J.A. & Loake, G.J. (2005) A central role for *S*‐nitrosothiols in plant disease resistance. Proceedings of the National Academy of Sciences of the United States of America, 102, 8054–8059. 15911759 10.1073/pnas.0501456102 PMC1142375

[mpp13102-bib-0013] Fisher, M.C., Henk, D.A., Briggs, C.J., Brownstein, J.S., Madoff, L.C., McCraw, S.L. et al. (2012) Emerging fungal threats to animal, plant and ecosystem health. Nature, 484, 186. 22498624 10.1038/nature10947 PMC3821985

[mpp13102-bib-0014] Floryszak‐Wieczorek, J., Arasimowicz‐Jelonek, M. & Izbiańska, K. (2016) The combined nitrate reductase and nitrite‐dependent route of NO synthesis in potato immunity to *Phytophthora infestans* . Plant Physiology and Biochemistry, 108, 468–477. 27588710 10.1016/j.plaphy.2016.08.009

[mpp13102-bib-0015] Foissner, I., Wendehenne, D., Langebartels, C. & Durner, J. (2000) In vivo imaging of an elicitor‐induced nitric oxide burst in tobacco. The Plant Journal, 23, 817–824. 10998192 10.1046/j.1365-313x.2000.00835.x

[mpp13102-bib-0016] Frungillo, L., Skelly, M.J., Loake, G.J., Spoel, S.H. & Salgado, I. (2014) *S*‐nitrosothiols regulate nitric oxide production and storage in plants through the nitrogen assimilation pathway. Nature Communications, 5, 5401. 10.1038/ncomms6401 PMC4229994 25384398

[mpp13102-bib-0017] Gong, B., Yan, Y., Zhang, L., Cheng, F., Liu, Z. & Shi, Q. (2019) Unravelling GSNOR‐mediated *S*‐nitrosylation and multiple developmental programs in tomato plants. Plant and Cell Physiology, 60, 2523–2537. 31350547 10.1093/pcp/pcz143

[mpp13102-bib-0018] Grant, J.J. & Loake, G.J. (2000) Role of reactive oxygen intermediates and cognate redox signaling in disease resistance. Plant Physiology, 124, 21–30. 10982418 10.1104/pp.124.1.21 PMC1539275

[mpp13102-bib-0019] Grünwald, N.J., Garbelotto, M., Goss, E.M., Heungens, K. & Prospero, S. (2012) Emergence of the sudden oak death pathogen *Phytophthora ramorum* . Trends in Microbiology, 20, 131–138. 22326131 10.1016/j.tim.2011.12.006

[mpp13102-bib-0020] Hussain, A., Yun, B.‐W., Kim, J.H., Gupta, K.J., Hyung, N.‐I. & Loake, G.J. (2019) Novel and conserved functions of *S*‐nitrosoglutathione reductase in tomato. Journal of Experimental Botany, 70, 4877–4886. 31089684 10.1093/jxb/erz234 PMC6760305

[mpp13102-bib-0021] Ichinose, Y., Shimizu, R., Ikeda, Y., Taguchi, F., Marutani, M., Mukaihara, T. et al. (2003) Need for flagella for complete virulence of *Pseudomonas syringae* pv. *tabaci*: genetic analysis with flagella‐defective mutants Δ*fliC* and Δ*fliD* in host tobacco plants. Journal of General Plant Pathology, 69, 244–249.

[mpp13102-bib-0023] Jaffrey, S.R. & Snyder, S.H. (2001) The biotin switch method for the detection of *S*‐nitrosylated proteins. Science's STKE, 2001, pl1. 10.1126/stke.2001.86.pl1 11752655

[mpp13102-bib-0024] Jahnová, J., Luhová, L. & Petřivalský, M. (2019) *S*‐nitrosoglutathione reductase‐the master regulator of protein S‐nitrosation in plant NO signaling. Plants, 8, 48. 10.3390/plants8020048 PMC6409631 30795534

[mpp13102-bib-0025] Jiang, R.H. & Tyler, B.M. (2012) Mechanisms and evolution of virulence in oomycetes. Annual Review of Phytopathology, 50, 295–318. 10.1146/annurev-phyto-081211-172912 22920560

[mpp13102-bib-0026] Jones, J.D. & Dangl, J.L. (2006) The plant immune system. Nature, 444, 323. 17108957 10.1038/nature05286

[mpp13102-bib-0027] Jung, T., Orlikowski, L., Henricot, B., Abad‐Campos, P., Aday, A., Aguín Casal, O. et al. (2016) Widespread *Phytophthora infestations* in European nurseries put forest, semi‐natural and horticultural ecosystems at high risk of *Phytophthora* diseases. Forest Pathology, 46, 134–163.

[mpp13102-bib-0028] Kamoun, S., Furzer, O., Jones, J.D.G., Judelson, H.S., Ali, G.S., Dalio, R.J.D. et al. (2015) The top 10 oomycete pathogens in molecular plant pathology. Molecular Plant Pathology, 16, 413–434. 25178392 10.1111/mpp.12190 PMC6638381

[mpp13102-bib-0029] Kamoun, S., Young, M., Glascock, C.B. & Tyler, B.M. (1993) Extracellular protein elicitors from *Phytophthora*: host‐specificity and induction of resistance to bacterial and fungal phytopathogens. Molecular Plant‐Microbe Interactions, 6, 15–25.

[mpp13102-bib-0030] Kwon, E., Feechan, A., Yun, B.‐W., Hwang, B.‐H., Pallas, J.A., Kang, J.‐G. et al. (2012) AtGSNOR1 function is required for multiple developmental programs in *Arabidopsis* . Planta, 236, 887–900. 22767201 10.1007/s00425-012-1697-8

[mpp13102-bib-0031] Latijnhouwers, M., de Wit, P.J. & Govers, F. (2003) Oomycetes and fungi: similar weaponry to attack plants. Trends in Microbiology, 11, 462–469. 14557029 10.1016/j.tim.2003.08.002

[mpp13102-bib-0032] Lee, U., Wie, C., Fernandez, B.O., Feelisch, M. & Vierling, E. (2008) Modulation of nitrosative stress by *S*‐nitrosoglutathione reductase is critical for thermotolerance and plant growth in *Arabidopsis* . The Plant Cell, 20, 786–802. 18326829 10.1105/tpc.107.052647 PMC2329944

[mpp13102-bib-0033] Li, H., Culligan, K., Dixon, R.A. & Chory, J. (1995) CUE1: a mesophyll cell‐specific positive regulator of light‐controlled gene expression in *Arabidopsis* . The Plant Cell, 7, 1599–1610. 12242356 10.1105/tpc.7.10.1599 PMC161018

[mpp13102-bib-0034] Li, Q., Ai, G., Shen, D., Zou, F., Wang, J., Bai, T. et al. (2019) A *Phytophthora capsici* effector targets ACD11 binding partners that regulate ROS‐mediated defense response in *Arabidopsis* . Molecular Plant, 12, 565–581. 30703564 10.1016/j.molp.2019.01.018

[mpp13102-bib-0035] Li, Q., Chen, Y., Wang, J., Zou, F., Jia, Y., Shen, D. et al. (2019) A *Phytophthora capsici* virulence effector associates with NPR1 and suppresses plant immune responses. Phytopathology Research, 1, 6.

[mpp13102-bib-0036] Li, Q., Wang, J., Bai, T., Zhang, M., Jia, Y., Shen, D. et al. (2020) A *Phytophthora capsici* effector suppresses plant immunity via interaction with EDS1. Molecular Plant Pathology, 21, 502–511. 31997517 10.1111/mpp.12912 PMC7060136

[mpp13102-bib-0037] Li, W., Zhao, D., Dong, J., Kong, X., Zhang, Q., Li, T. et al. (2020) *AtRTP5* negatively regulates plant resistance to *Phytophthora* pathogens by modulating the biosynthesis of endogenous jasmonic acid and salicylic acid. Molecular Plant Pathology, 21, 95–108. 31701600 10.1111/mpp.12883 PMC6913198

[mpp13102-bib-0038] Li, Q., Zhang, M., Shen, D., Liu, T., Chen, Y., Zhou, J.‐M. et al. (2016) A *Phytophthora sojae* effector PsCRN63 forms homo‐/hetero‐dimers to suppress plant immunity via an inverted association manner. Scientific Reports, 6, 26951. 27243217 10.1038/srep26951 PMC4886637

[mpp13102-bib-0039] Lindermayr, C., Sell, S., Müller, B., Leister, D. & Durner, J. (2010) Redox regulation of the NPR1‐TGA1 system of *Arabidopsis thaliana* by nitric oxide. The Plant Cell, 22, 2894–2907. 20716698 10.1105/tpc.109.066464 PMC2947166

[mpp13102-bib-0040] Ling, T., Bellin, D., Vandelle, E., Imanifard, Z. & Delledonne, M. (2017) Host‐mediated *S*‐nitrosylation disarms the bacterial effector HopAI1 to reestablish immunity. The Plant Cell, 29, 2871–2881. 29084872 10.1105/tpc.16.00557 PMC5728119

[mpp13102-bib-0041] Liu, T., Song, T., Zhang, X., Yuan, H., Su, L., Li, W. et al. (2014) Unconventionally secreted effectors of two filamentous pathogens target plant salicylate biosynthesis. Nature Communications, 5, 4686. 10.1038/ncomms5686 PMC4348438 25156390

[mpp13102-bib-0042] Matamoros, M.A., Cutrona, M.C., Wienkoop, S., Begara‐Morales, J.C., Sandal, N., Orera, I. et al. (2020) Altered plant and nodule development and protein S‐nitrosylation in *Lotus japonicus* mutants deficient in S‐nitrosoglutathione reductases. Plant and Cell Physiology, 61, 105–117. 31529085 10.1093/pcp/pcz182

[mpp13102-bib-0043] Modolo, L.V., Augusto, O., Almeida, I.M., Pinto‐Maglio, C.A., Oliveira, H.C., Seligman, K. et al. (2006) Decreased arginine and nitrite levels in nitrate reductase‐deficient *Arabidopsis thaliana* plants impair nitric oxide synthesis and the hypersensitive response to *Pseudomonas syringae* . Plant Science, 171, 34–40.

[mpp13102-bib-0044] Nowicki, M., Foolad, M.R., Nowakowska, M. & Kozik, E.U. (2012) Potato and tomato late blight caused by *Phytophthora infestans*: an overview of pathology and resistance breeding. Plant Disease, 96, 4–17. 30731850 10.1094/PDIS-05-11-0458

[mpp13102-bib-0045] Pan, Q., Cui, B., Deng, F., Quan, J., Loake, G.J. & Shan, W. (2016) *RTP1* encodes a novel endoplasmic reticulum (ER)‐localized protein in *Arabidopsis* and negatively regulates resistance against biotrophic pathogens. New Phytologist, 209, 1641–1654. 10.1111/nph.13707 26484750

[mpp13102-bib-0046] Requena, M.E., Egea‐Gilabert, C. & Candela, M.E. (2005) Nitric oxide generation during the interaction with *Phytophthora capsici* of two *Capsicum annuum* varieties showing different degrees of sensitivity. Physiologia Plantarum, 124, 50–60.

[mpp13102-bib-0047] Shibata, Y., Kawakita, K. & Takemoto, D. (2010) Age‐related resistance of *Nicotiana benthamiana* against hemibiotrophic pathogen *Phytophthora infestans* requires both ethylene‐and salicylic acid–mediated signaling pathways. Molecular Plant‐Microbe Interactions, 23, 1130–1142. 20687803 10.1094/MPMI-23-9-1130

[mpp13102-bib-0048] Tada, Y., Spoel, S.H., Pajerowska‐Mukhtar, K., Mou, Z., Song, J., Wang, C. et al. (2008) Plant immunity requires conformational charges of NPR1 via *S*‐nitrosylation and thioredoxins. Science, 321, 952–956. 18635760 10.1126/science.1156970 PMC3833675

[mpp13102-bib-0049] Tichá, T., Sedlářová, M., Činčalová, L., Trojanová, Z.D., Mieslerová, B., Lebeda, A. et al. (2018) Involvement of S‐nitrosothiols modulation by *S*‐nitrosoglutathione reductase in defence responses of lettuce and wild *Lactuca* spp. to biotrophic mildews. Planta, 247, 1203–1215. 29417270 10.1007/s00425-018-2858-1

[mpp13102-bib-0067] Thordal‐Christensen, H., Zhang, Z., Wei, Y. & Collinge, D.B. (1997) Subcellular localization of H_2_O_2_ in plants. H_2_O_2_ accumulation in papillae and hypersensitive response during the barley–powdery mildew interaction. The Plant Journal, 11, 1187–1194.

[mpp13102-bib-0050] Torres, M.A., Dangl, J.L. & Jones, J.D. (2002) *Arabidopsis* gp91^phox^ homologues *AtrbohD* and *AtrbohF* are required for accumulation of reactive oxygen intermediates in the plant defense response. Proceedings of the National Academy of Sciences of the United States of America, 99, 517–522. 11756663 10.1073/pnas.012452499 PMC117592

[mpp13102-bib-0051] Umbreen, S., Lubega, J., Cui, B., Pan, Q., Jiang, J. & Loake, G.J. (2018) Specificity in nitric oxide signalling. Journal of Experimental Botany, 69, 3439–3448. 29767796 10.1093/jxb/ery184

[mpp13102-bib-0052] Van De Peer, Y. & de Wachter, R. (1997) Evolutionary relationships among the eukaryotic crown taxa taking into account site‐to‐site rate variation in 18S rRNA. Journal of Molecular Evolution, 45, 619–630. 9419239 10.1007/pl00006266

[mpp13102-bib-0054] Wang, Y., Bouwmeester, K., Beseh, P., Shan, W. & Govers, F. (2014) Phenotypic analyses of *Arabidopsis* T‐DNA insertion lines and expression profiling reveal that multiple L‐type lectin receptor kinases are involved in plant immunity. Molecular Plant‐Microbe Interactions, 27, 1390–1402. 25083911 10.1094/MPMI-06-14-0191-R

[mpp13102-bib-0055] Wang, Y., Feechan, A., Yun, B.‐W., Shafiei, R., Hofmann, A., Taylor, P. et al. (2009) *S*‐nitrosylation of AtSABP3 antagonizes the expression of plant immunity. Journal of Biological Chemistry, 284, 2131–2137. 10.1074/jbc.M806782200 19017644

[mpp13102-bib-0056] Wang, Y., Meng, Y., Zhang, M., Tong, X., Wang, Q., Sun, Y. et al. (2011) Infection of *Arabidopsis thaliana* by *Phytophthora parasitica* and identification of variation in host specificity. Molecular Plant Pathology, 12, 187–201. 21199568 10.1111/j.1364-3703.2010.00659.x PMC6640465

[mpp13102-bib-0057] Wang, Y., Tyler, B.M. & Wang, Y. (2019) Defense and counterdefense during plant‐pathogenic oomycete infection. Annual Review of Microbiology, 73, 667–696. 10.1146/annurev-micro-020518-120022 31226025

[mpp13102-bib-0058] Wi, S.J., Ji, N.R. & Park, K.Y. (2012) Synergistic biosynthesis of biphasic ethylene and reactive oxygen species in response to hemibiotrophic *Phytophthora parasitica* in tobacco plants. Plant Physiology, 159, 251–265. 22388490 10.1104/pp.112.194654 PMC3375963

[mpp13102-bib-0059] Wilkinson, J.Q. & Crawford, N.M. (1993) Identification and characterization of a chlorate‐resistant mutant of *Arabidopsis thaliana* with mutations in both nitrate reductase structural genes *NIA1* and *NIA2* . Molecular and General Genetics, 239, 289–297. 8510658 10.1007/BF00281630

[mpp13102-bib-0060] Yang, B., Wang, Q., Jing, M., Guo, B., Wu, J., Wang, H. et al. (2017) Distinct regions of the *Phytophthora* essential effector Avh238 determine its function in cell death activation and plant immunity suppression. New Phytologist, 214, 361–375. 10.1111/nph.14430 28134441

[mpp13102-bib-0061] Yu, M., Lamattina, L., Spoel, S.H. & Loake, G.J. (2014) Nitric oxide function in plant biology: a redox cue in deconvolution. New Phytologist, 202, 1142–1156. 10.1111/nph.12739 24611485

[mpp13102-bib-0062] Yun, B.‐W., Feechan, A., Yin, M., Saidi, N.B., le Bihan, T. , Yu, M. et al. (2011) S‐nitrosylation of NADPH oxidase regulates cell death in plant immunity. Nature, 478, 264. 21964330 10.1038/nature10427

[mpp13102-bib-0063] Yun, B.W., Skelly, M.J., Yin, M., Yu, M., Mun, B.G., Lee, S.U. et al (2016) Nitric oxide and *S*‐nitrosoglutathione function additively during plant immunity. New Phytologist, 211, 516–526. 10.1111/nph.13903 26916092

[mpp13102-bib-0064] Zhang, M., Li, Q., Liu, T., Liu, L., Shen, D., Zhu, Y. et al. (2015) Two cytoplasmic effectors of *Phytophthora sojae* regulate plant cell death via interactions with plant catalases. Plant Physiology, 167, 164–175. 25424308 10.1104/pp.114.252437 PMC4281015

[mpp13102-bib-0065] Zhang, Y. & Li, X. (2019) Salicylic acid: biosynthesis, perception, and contributions to plant immunity. Current Opinion in Plant Biology, 50, 29–36. 30901692 10.1016/j.pbi.2019.02.004

[mpp13102-bib-0066] Zheng, X., McLellan, H., Fraiture, M., Liu, X., Boevink, P.C., Gilroy, E.M. et al. (2014) Functionally redundant RXLR effectors from *Phytophthora infestans* act at different steps to suppress early flg22‐triggered immunity. PLoS Pathogens, 10, e1004057. 24763622 10.1371/journal.ppat.1004057 PMC3999189

