# Supplementary material for: Perturbations in nitric oxide homeostasis promote Arabidopsis disease susceptibility towards Phytophthora parasitica
Source: Mol Plant Pathol. 2021 Jul 9;22(9):1134–48. doi: 10.1111/mpp.13102 (PMC8359001; doi:10.1111/mpp.13102)
Supplement: Supplementary file 1 — FIGURE S1 Reduced NO levels affect Arabidopsis resistance to Phytophthora parasitica [file MPP-22-1134-s004.docx]

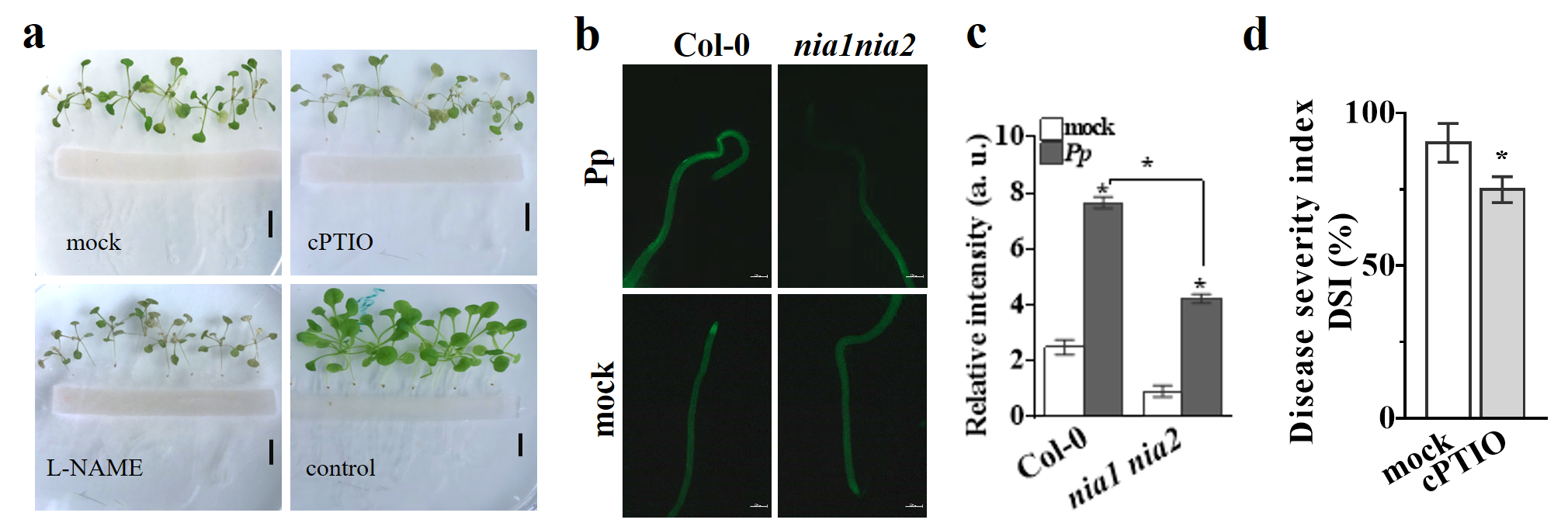


**Fig. S1 Reduced NO levels affect *Arabidopsis* resistance to *P. parasitica*.**

(a) Phenotype of *Arabidopsis* seedlings upon *P. parasitica* inoculation at 4 dpi by adding indicated compound. Water was added as mock, V8 plug without pathogen was used as control.

(b-c) NO levels of *nia1nia2* were determined by DAF-FM (b) and intensity of DAF-FM staining was established by Image J software (c). Error bars represent SD of 8 replicates.

(d) Disease survey index of *nox1* upon *P. parasitica* inoculation at 4 dpi treated with cPTIO. Error bars represent SD of 3 biological replicates, 8 seedlings were used for each experiment. *****, *P* value< 0.05.
