# Supplementary material for: Perturbations in nitric oxide homeostasis promote Arabidopsis disease susceptibility towards Phytophthora parasitica
Source: Mol Plant Pathol. 2021 Jul 9;22(9):1134–48. doi: 10.1111/mpp.13102 (PMC8359001; doi:10.1111/mpp.13102)
Supplement: Supplementary file 2 — FIGURE S2GSNOR1 loss‐of‐function mutants are more susceptible to Phytophthora parasitica infection [file MPP-22-1134-s001.docx]

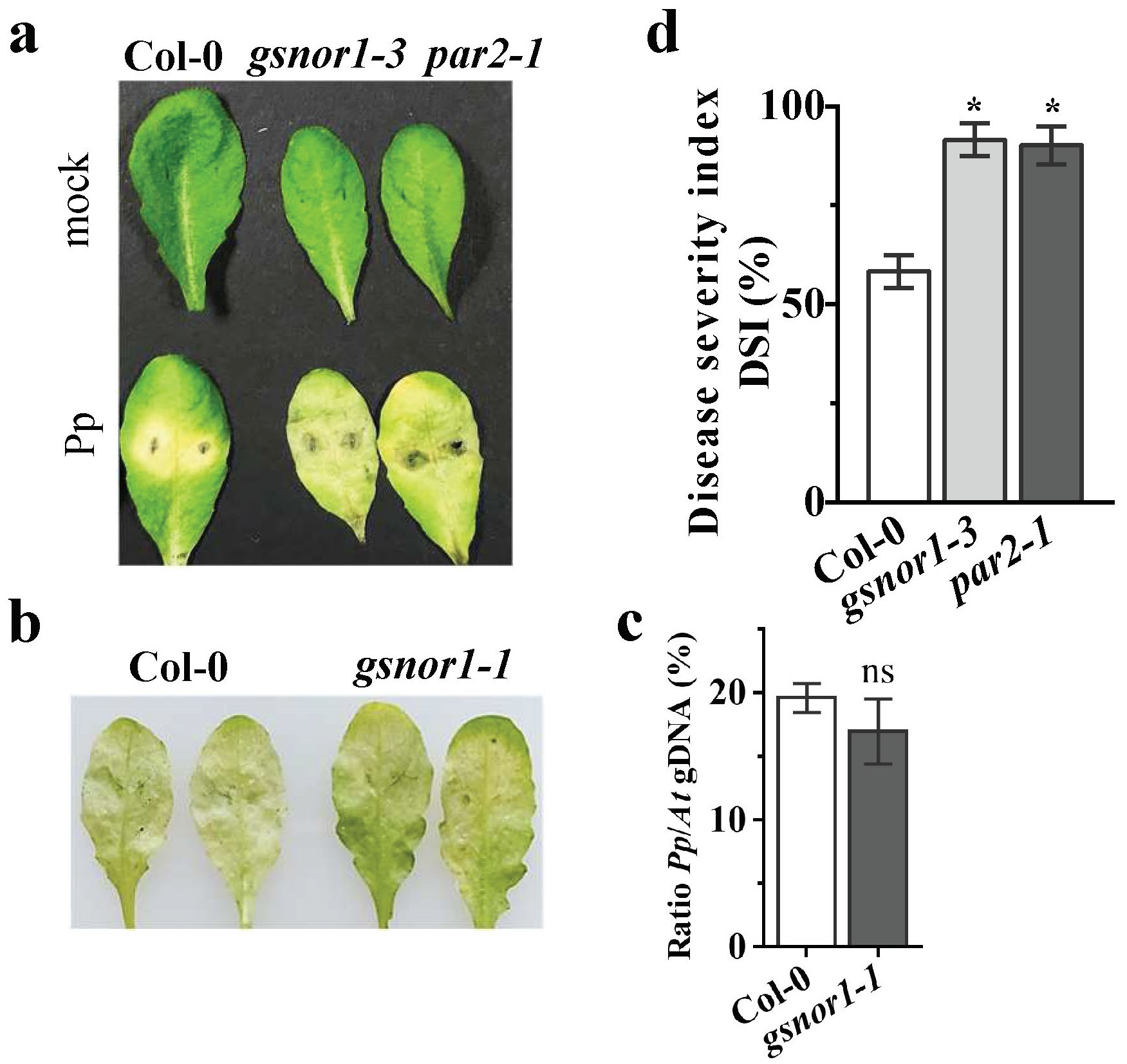


**Fig. S2 *GSNOR1* loss-of-function mutants are more susceptible to *P. parasitica* infection**

(a) Phenotype of indicated plants 3 days post *P. parasitica* infection (1 x 10^5^ zoospores).

(b) Phenotype of GSNOR overexpression transgenic plant upon *P. parasitica* infection 3 dpi.

(c) Pathogen biomass analysis by qPCR for (c) after *P. parasitica* inoculation. Error bars indicate SD of three biological replicates. ns, no significant difference, *t* test, *p*<0.05.

(d) Disease survey index of *GSNOR1* mutant seedlings inoculated upon *P. parasitica* inoculation was analyzed at 4 dpi. 16 seedlings were used for each plant line. Error bars indicate ± SD of three biological replicates. ***, *P* < 0.001.
