# Supplementary material for: Perturbations in nitric oxide homeostasis promote Arabidopsis disease susceptibility towards Phytophthora parasitica
Source: Mol Plant Pathol. 2021 Jul 9;22(9):1134–48. doi: 10.1111/mpp.13102 (PMC8359001; doi:10.1111/mpp.13102)
Supplement: Supplementary file 3 — FIGURE S3 Protein stability assay after GSNOR activity assay [file MPP-22-1134-s002.docx]

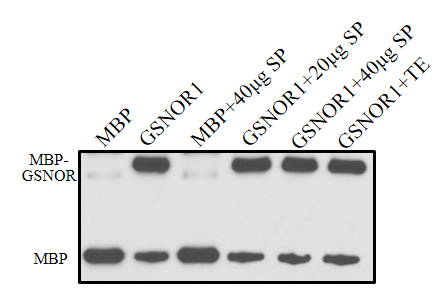


**Fig. S3 Protein stability assay after GSNOR activity assay.**

The samples after GSNOR activity assay in Fig. 7a were loaded to SDS-PAGE following by western blot detected by MBP antibody (Anti-MBP Monoclonal Antibody, #E8032, NEB) to rule out the possibility of protein degradation.
