# Supplementary material for: Perturbations in nitric oxide homeostasis promote Arabidopsis disease susceptibility towards Phytophthora parasitica
Source: Mol Plant Pathol. 2021 Jul 9;22(9):1134–48. doi: 10.1111/mpp.13102 (PMC8359001; doi:10.1111/mpp.13102)
Supplement: Supplementary file 4 — TABLE S1 Primer list [file MPP-22-1134-s003.docx]

**Table S1 Primer list.**

| **Primers for qRT-PCR** | | |
| --- | --- | --- |
| GSNOR1-FW | | TACTGTTGTTCATGATGTTAGCGTCG |
| GSNOR1-RV | | TGCTATCGATGTCAATGCCAATG |
| UBQ10-FW | | AGATCCAGGACAAGGAAGGTATTC |
| UBQ10-RV | | CGCAGGACCAAGTGAAGAGTAG |
| PR1-FW | | CGTTCACATAATTCCCACGAG |
| PR1-RV | | TCAGTGAGACTCGGATGTGC |
| PR5-FW | GTTCATCACAAGCGGCATT |  |
| PR5-RV | GTCAATTCAAATCCTCCATCG |  |
| WRKY62-FW | | GTTTCTCAGATGCGCTCTCC |
| WRKY62-RV | | CTGGCCGAAGTAAGTGGTTG |
| ICS1-FW | | CCCTTAACAAGGTTGTTCTTGC |
| ICS1-RV | | CCTTCACGCTGTAACTGTGC |
| CBP60g-FW | | AAGAAGAATTGTCCGAGAGGAG |
| CBP60g-RV | | GGCGAGTTTATGAAGCACAG |
| PAD4-FW | | TCTTCAGTTAAAGATCAAGGAAGG |
| PAD4-RV | | GGTTGAATGGCCGGTTATC |
| RBOHD-FW | | GCCAAAGAATATGCGGCACT |
| RBOHD-RV | | TTGCTCTCTTTTGCCGGTCT |
| **Primers for biomass assay** | | |
| AtUBC9g-FW | | TTCATTGGCAGGCCACTAT |
| AtUBC9g-RV | | CTTAGGAGGCTTAAATGGGTAA |
| PpUBCg-FW | | CCACTTAGAGCACGCTAGGA |
| PpUBCg-RV | | TACCGACTGTCCTTCGTTCA |
